# How out-group animosity can shape partisan divisions: A model of affective polarization

**DOI:** 10.1093/pnasnexus/pgaf082

**Published:** 2025-03-11

**Authors:** Buddhika Nettasinghe, Allon G Percus, Kristina Lerman

**Affiliations:** Department of Business Analytics, Tippie College of Business, University of Iowa, Iowa City, IA 52242, USA; Institute of Mathematical Sciences, Claremont Graduate University, Claremont, CA 91711, USA; Information Sciences Institute, University of Southern California, Marina del Rey, CA 90292, USA

**Keywords:** affective polarization, opinion dynamics, social networks, homophily, political psychology

## Abstract

Politically divided societies are also often divided emotionally: people like and trust those with similar political views (in-group favoritism) while disliking and distrusting those with different views (out-group animosity). This phenomenon, called affective polarization, influences individual decisions, including seemingly apolitical choices such as whether to wear a mask or what car to buy. We present a dynamical model of decision-making in an affectively polarized society, identifying three potential global outcomes separated by a sharp boundary in the parameter space: consensus, partisan polarization, and nonpartisan polarization. Analysis reveals that larger out-group animosity compared to in-group favoritism, i.e. *more hate than love*, is sufficient for polarization, while larger in-group favoritism compared to out-group animosity, i.e. *more love than hate*, is necessary for consensus. We also show that, counterintuitively, increasing cross-party connections facilitates polarization, and that by emphasizing partisan differences, mass media creates self-fulfilling prophecies that lead to polarization. Affective polarization also creates *tipping points* in the opinion landscape where one group suddenly reverses their trends. Our findings aid in understanding and addressing the cascading effects of affective polarization, offering insights for strategies to mitigate polarization.

**Significance Statement**

The escalation of partisan divide threatens social cohesion and effective governance. This article presents a mathematical model showing how affective polarization—emotional animosity to the opposing party and affection towards one’s own party—can transcend ideology, driving rapid transitions between consensus, polarization, and fragmentation in collective choices. The model explains how out-group hate is a potent driver of division, while in-group love is less strong as a unifier, highlighting the challenges of finding compromise in a divided society. Counterintuitively, forcefully breaking echo chambers in societies with high animosity fuels polarization rather than deterring it. The analytically tractable model reconciles seemingly contradictory findings in the literature and provides a theoretical foundation to study and mitigate harmful polarization dynamics.

## Introduction

American society has grown more ideologically divided, with Democrats and Republicans not only disagreeing on policy issues but also making dramatically different choices about where to live and work, what products to buy, leisure activities to pursue (1), or sports teams to support (2). Surveys also reveal a growing emotional divide, with members of each party increasingly disliking and distrusting the opposing party (3, 4). This phenomenon, called affective polarization, is manifested in people expressing warm feelings, i.e. *in-group love*, towards their ideological allies but negative feelings and animosity, i.e. *out-group hate*, to members of the opposing party. Over the last decade, cross-party antipathy has grown and now exceeds in-group love (5, 6). The escalating partisan animosity poses a challenge to effective governing and the well-being of society. For example, during the COVID-19 pandemic individuals’ trust and adherence to public health recommendations, like wearing a mask or getting vaccinated, were shaped by whether their own political party supported or opposed those recommendations (7), hindering an effective response to the pandemic.

Research has shown that demographics alone cannot account for the partisan divide in beliefs and behaviors (8–10). Instead, these phenomena arise from collective social dynamics. The tendency to associate with others who are similar, a process known as homophily, amplifies chance correlations between individual preferences and ideology, giving rise to a unified behavior within a group over time. This effect was used to explain the emergence of stereotypes like “latte-drinking liberals” and “bird-hunting conservatives” (1). The rise of online media has further amplified social cleavages by enabling people to align their information environments with their ideology. Similar to the mechanisms described above, these preferences tend to segregate people within ideologically homogeneous communities, i.e. echo chambers (11, 12), which insulate them from opposing views and promote polarization. However, recent research has challenged this understanding (13), pointing to studies that show instead how increasing polarization can arise from exposure to opposing views.

This article presents a model of information cascades in an affectively polarized social network composed of two groups (e.g. red and blue), where individuals within each group like and trust members of their own group (in-group love) and dislike and distrust members of the other group (out-group hate).^a^ When choosing between two possible choices (e.g. wear a mask or not, get vaccinated or not, which team to support in the Superbowl), individuals observe their social connections and attempt to *conform* to the choices of their in-group and *oppose* choices made by members of their out-group. Depending on the size of the minority and majority groups, homophily (preference of individuals to connect to others of the same group), and the levels of in-group conformity and out-group opposition, several different long-term outcomes can emerge, marked by a sharp boundary: global consensus (all individuals adopt the same choice), polarization (party-line division of choices) and nonpartisan polarization in which each group’s choices are uniformly divided. We theoretically characterize the conditions under which such outcomes occur and provide numerical experiments and results using two real-world social network datasets that yield further insights.

Despite its simplicity, the model exhibits remarkably complex behaviors and reconciles seemingly contradictory findings from literature. The model explains how rapid collective transitions, or *tipping points* in the opinion landscape (15), can emerge in social systems. Even when both parties are close to reaching a compromise, the presence of such tipping points due to out-group hate has the potential to disrupt consensus, a pattern that is increasingly observed within emotionally polarized societies. It shows that opposition to the choices by members of the other party, driven by out-group hate, is a potent driver of polarization. When out-group hate is stronger than in-group love, no consensus is feasible. This may explain why disagreement on issues between Democrats and Republicans accelerated since 2012, when out-group hate exceeded in-group love in the United States (6). The model also explains why conventional wisdom-based approaches aimed at reducing polarization, such as connecting people from opposite parties, often backfire (13, 16). Specifically, our results corroborate the findings in Refs. (17–19) showing that consensus can be achieved only when antagonistic communities are loosely connected and in the absence of contrarian agents. The model illustrates that the mere existence of people’s desire to be similar to the in-group (in-group love) and different from out-group (out-group hate) alone cannot fully explain the emergence of polarization; consensus can emerge even in the presence of such emotional divides. Going beyond, our analysis provides a comprehensive explanation for the role of out-group hate, in-group love, group sizes, cross-party connections, and initial beliefs in shaping opinions. Our work suggests that emphasizing partisan differences, even when they are small, can fuel polarization through a self-fulfilling prophecy. To counteract this, news media and social platforms could instead strive to diminish the perception of party-line differences to impede actual polarization. For example, our model theoretically explains why exposure to similar individuals from opposing parties may be one of the few effective methods to facilitate consensus in an affectively polarized society (20).

Although our model is parameterized by only two key quantities, it replicates a wide range of real-world phenomena and leads to new insights into polarization, as well as methods to mitigate it. The theoretical tractability of the model, which yields closed-form expressions for its dynamics, reduces the need to rely on large scale simulations to obtain such insights and may lead to new solutions to control polarization. Easy implementation on any arbitrary network also facilitates the study of affective polarization on synthetic and real-world networks.

Compared to existing models of opinion dynamics, the model we propose has three key differences: explicit parametrization of the in-group love and out-group hate, a bi-populated society (with two opposing parties), and binary decisions. A theoretically tractable model that integrates all three of these essential characteristics of affective polarization has remained a gap in the literature. Models with a continuous decision (opinion) variable (e.g. DeGroot type models (21), Altafani model (22), etc.) are not optimal for capturing the inherently binary nature of choices that end up being polarized along party lines (e.g. wear a mask or not, vaccinate or not (23, 24)). Models that have a binary decision variable (e.g. independent cascade model, linear threshold model (25)) do not explicitly account for the affective polarization via in-group love and out-group hate in a bipopulated society. Despite being highly useful in understanding homogeneous populations composed of friends only, such models are not adequate for exploring affective polarization in bi-populated societies. While there have been models specifically aimed at understanding the emergence of affective polarization (e.g. (1, 13)), they do not provide an explicit parameterization of the two characteristic features of affective polarization, namely in-group love and out-group hate, or the theoretical tractability that yields closed-form expressions.

## A model of information cascades with affective polarization

We present a dynamical model of how people make choices in a social network (e.g. to mask or support a sports team) by viewing the past choices of their in-group (e.g. members of their own party), which they approve of, as well as the choices of their out-group (e.g. cross-party members), which they oppose. The choice dynamics lead to an information cascade which reaches a steady state of partisan polarization or consensus depending on group sizes and the levels of in-group love and out-group hate.

Consider an undirected social network G=(V,E) with N=|V| individuals. Each individual (node) v∈V has two binary attributes: a static binary attribute R(v)∈{0,1} and a dynamic binary attribute $H_{k}(v)\in \{0,1\}$, where *k* denotes discrete-time. The static attribute represents the group (e.g. political) affiliation: *v* is red (v∈R) if R(v)=1; otherwise, *v* is blue (v∈B). Let $N^{B}=|B|$ and $N^{R}=|R|$ denote the sizes of the two groups and $r=N^{R}/N$ denote the fraction of red nodes. The dynamic attribute $H_{k}(v)\in \{0,1\}$ represents *v*’s choice at time *k* (e.g. wearing a mask vs not wearing a mask).

At each time *k* (where k=0,1,2,…), a node $X_{k}\in V$ chosen uniformly at random updates its choice by observing the choices of its neighbors. Let


(1) $$\begin{array}{cc} d_{k}^{\mathrm{in},0}(X_{k}) & =\sum_{(X_{k},u)\in E}1(R(u)=R(X_{k})\wedge H_{k}(u)=0)/d(X_{k})\\ d_{k}^{\mathrm{in},1}(X_{k}) & =\sum_{(X_{k},u)\in E}1(R(u)=R(X_{k})\wedge H_{k}(u)=1)/d(X_{k})\\ d_{k}^{\mathrm{out},0}(X_{k}) & =\sum_{(X_{k},u)\in E}1(R(u)\neq R(X_{k})\wedge H_{k}(u)=0)/d(X_{k})\\ d_{k}^{\mathrm{out},1}(X_{k}) & =\sum_{(X_{k},u)\in E}1(R(u)\neq R(X_{k})\wedge H_{k}(u)=1)/d(X_{k}) \end{array}$$

denote the number of in-group and out-group neighbors with choice-0 and choice-1 at time *k* normalized by the total number of neighbors $d(X_{k})$. Node $X_{k}$ updates its choice at k+1 according to:


(2) $$H_{k+1}(X_{k})=\left\{ \begin{array}{cc} 0 & \;\text{if }\alpha \left(d_{k}^{\mathrm{in},1}(X_{k})-d_{k}^{\mathrm{in},0}(X_{k})\right)-\beta \left(d_{k}^{\mathrm{out},1}(X_{k})-d_{k}^{\mathrm{out},0}(X_{k})\right)<-\delta \\ 1 & \;\text{if }\alpha \left(d_{k}^{\mathrm{in},1}(X_{k})-d_{k}^{\mathrm{in},0}(X_{k})\right)-\beta \left(d_{k}^{\mathrm{out},1}(X_{k})-d_{k}^{\mathrm{out},0}(X_{k})\right)>\delta \\ H_{k}(X_{k}) & \text{otherwise}, \end{array}\right.$$

where α,β,δ∈[0,1] are constant model parameters. Choices of all other nodes except $X_{k}\in V$ remain unchanged: for all $u\neq X_{k},H_{k+1}(u)=H_{k}(u)$.

The above stylized model aims to capture the dynamics of choices in an affectively polarized society. To explain the intuition behind the model, let us consider masking as the dynamic attribute.^b^ Consider a red node *v* deciding whether to wear a mask during the pandemic. The red neighbors (in-group) that wear masks push *v* towards masking, whereas the red neighbors who do not wear masks push *v* towards not-masking. The out-group (blue) neighbors have the opposite effect: blue masking neighbors push node *v* towards not-masking, whereas blue nonmasking neighbors push the node towards masking. The relative strengths of these effects, *in-group love* and *out-group hate*, are quantified by *α* and *β*, respectively.^c^ If the combined effect of out-group hate and in-group love exceeds *δ* in favor of a certain choice (1 or 0), then *v* adopts it. If not, it keeps it current choice. Thus, *δ* quantifies the level of *inertia* of a person, or the degree of social proof, including from the out-group, required to change the choice. Also note from Eq. 2 that, among the neighbors of *v* belonging to each group, only the difference between how many chose choice-0 and choice-1 matters and not the ratio. Even with the normalization in Eq. 1, 50 out of a total of 100 masking blue neighbors will create a greater out-group effect for a red node than when one out of two blue neighbors masks.

To analyze the dynamics, we examine the fraction of nodes in each group that have adopted choice-1 at time *k*. Formally, we define the state of the system at time *k* as the column vector $\theta_{k}=[\theta_{k}^{B},\theta_{k}^{R}{]}^{\prime }$ where,


(3) $$\theta_{k}^{B}=\frac{\sum_{v\in V}1(R(v)=0\wedge H_{k}(v)=1)}{\sum_{v\in V}1(R(v)=0)},\;\theta_{k}^{R}=\frac{\sum_{v\in V}1(R(v)=1\wedge H_{k}(v)=1)}{\sum_{v\in V}1(R(v)=1)}.$$

Since the node $X_{k}$ is chosen randomly at time *k* to update its choice, the trajectory of the system $\theta_{k}=[\theta_{k}^{B},\theta_{k}^{R}{]}^{\prime },k=0,1,2,\ldots$ is also a random process. We show that the discrete-time stochastic trajectory $\theta_{k},k=0,1,2,\ldots$ can be approximated using the continuous-time deterministic trajectory of a differential equation under a few assumptions. This differential equation representation of the stochastic model, called the *limit mean differential equation* can thus be used to analyze the emergence of various patterns in the social network over sufficiently large time horizons. We will focus on two cases of practical interest: a fully connected network and a stochastic block model.

### Dynamics of the model in a fully connected network

We first consider a fully connected social network G=(V,E), where each node v∈V can observe the state of the system $\theta_{k}=[\theta_{k}^{B},\theta_{k}^{R}{]}^{\prime }$ at any time *k*. This occurs, for example, when people are informed about the prevalence of masking within each political party via daily news broadcasts and make their decisions to mask accordingly.

In such a graph, the piece-wise interpolation^d^ of the discrete-time trajectory $\theta_{k}=[\theta_{k}^{B},\theta_{k}^{R}{]}^{\prime },k=0,1,2,\ldots$ can be approximated using the continuous-time trajectory $\theta (t)=[\theta^{B}(t),\theta^{R}(t){]}^{\prime },t\geq 0$ of the following differential equation as the number of nodes in the graph *N* is large:


(4) $$\left[\begin{array}{c} {\dot{\theta }}^{B}\\ {\dot{\theta }}^{R} \end{array}\right]=\left[\begin{array}{c} \left(1-\theta^{B}\right)p_{\theta }^{B}(0\to 1)-\theta^{B}p_{\theta }^{B}(1\to 0)\\ \left(1-\theta^{R}\right)p_{\theta }^{R}(0\to 1)-\theta^{R}p_{\theta }^{R}(1\to 0) \end{array}\right],$$

where,


$$\begin{array}{rl} & p_{\theta }^{B}(0\to 1)=1\left(\alpha (1-r)\left(2\theta^{B}-1\right)-\beta r\left(2\theta^{R}-1\right)>\delta \right),\\ & p_{\theta }^{B}(1\to 0)=1\left(\alpha (1-r)\left(2\theta^{B}-1\right)-\beta r\left(2\theta^{R}-1\right)<-\delta \right),\\ & p_{\theta }^{R}(0\to 1)=1\left(\alpha \left(2\theta^{R}-1\right)-\beta (1-r)\left(2\theta^{B}-1\right)>\delta \right),\\ & p_{\theta }^{R}(1\to 0)=1\left(\alpha \left(2\theta^{R}-1\right)-\beta (1-r)\left(2\theta^{B}-1\right)<-\delta \right). \end{array}$$

The intuition behind the differential equation in Eq. 4 is as follows. In a fully connected network, each node is a neighbor of all other nodes. Thus, the node-level statistics in Eq. 1 can be written using the population statistics in Eq. 3. For a blue node $X_{k}$, we can write $d_{k}^{\mathrm{in},1}(X_{k})=\theta_{k}^{B}(1-r),d_{k}^{\mathrm{in},0}(X_{k})=(1-\theta_{k}^{B})(1-r),d_{k}^{\mathrm{out},1}(X_{k})$  $=\theta_{k}^{R}r,d_{k}^{\mathrm{out},0}(X_{k})=(1-\theta_{k}^{R})r$. According to Eq. 2, a blue node $X_{k}$ picks choice-1 when $\alpha (1-r)(2\theta_{k}^{B}-1)-\beta (2\theta_{k}^{R}-1)>\delta$, i.e. positive influence from the presence of choice-1 among in-group neighbors is larger than the negative influence from the presence of choice-1 among out-group neighbors by a margin of at least *δ*. Similarly, a blue node picks choice-0 when $\alpha (1-r)(2\theta_{k}^{B}-1)-\beta (2\theta_{k}^{R}-1)<-\delta$. Since a fraction $1-\theta_{k}^{B}$ of blue nodes have choice-0 and a fraction $\theta_{k}^{B}$ of blue nodes have choice-1, the expected rate of change of blue nodes with choice-1 $\theta_{k}^{B}$ can thus be written as ${\dot{\theta }}^{B}$ in Eq. 4, and similarly for ${\dot{\theta }}^{R}$. When the network is large, the stochastic dynamics converge to the deterministic differential equation in Eq. 4. The formal proof of convergence (which uses tools from stochastic approximation theory (28) and discontinuous dynamical systems (29)) is given in Supplementary material, Section A. Thus, for any initial state $\theta (0)=[\theta^{B}(0),\theta^{R}(0){]}^{\prime }$, the continuous-time trajectory $\theta (t)=\theta (0)+\int_{0}^{t}\dot{\theta }(s)ds,t\geq 0$ obtained using Eq. 4 approximates the stochastic model dynamics $\theta_{k}=[\theta_{k}^{B},\theta_{k}^{R}{]}^{\prime },k=0,1,2,\ldots$.

In the remainder of the article, we use Eq. 4 and its generalizations to explore how polarized information cascades emerge in affectively polarized populations.

### Dynamics of the model on a social network with communities

Next, we consider the case where the network G=(V,E) is sampled from a stochastic block model with two communities. Specifically, each node is connected to a node in the same party with probability *ρ* and a node in the other party with probability 1−ρ, where ρ∈(0,1) is a constant model parameter. Thus, *ρ* quantifies the level of *homophily* (30) of the individuals in the population: ρ>0.5 implies that individuals are more likely to connect with others of the same party (homophily), whereas ρ<0.5 implies that individuals tend to mostly connect with members of the other party (heterophily). When ρ=0.5, the graph can be viewed as an Erdos–Rényi random graph with each edge being formed with a probability of 0.5.

Alternatively, *ρ* can be interpreted in the following way: each individual looks at a fraction *ρ* of their in-group members and a fraction 1−ρ of their out-group members and makes a decision based on their choices. Thus, *ρ* might also be used to represent the balance of information an individual receives from the news media in terms of how well they represent the two parties: ρ>0.5 means the news consumed by an individual over-represents views of the in-group (relative to its size), while ρ<0.5 means that the news over-represents the views of the out-group (relative to its size). When ρ=0.5, each group is represented in the news proportionate to its group size.

The dynamics of the system $\theta_{k}=[\theta_{k}^{B},\theta_{k}^{R}{]}^{\prime },k=0,1,2,\ldots$ in a stochastic block model network can be approximated using the continuous-time trajectory of Eq. 4 with *α* replaced by αρ and *β* replaced by β(1−ρ). In other words, the homophily *ρ* amplifies the effects of in-group love while reducing the effects of out-group hate. The exact differential equation for the stochastic block model is stated in Supplementary material, Section C.

## Results

We analyze dynamics of the model and obtain insights about information cascades in an affectively polarized society. We first focus on a fully connected population with no inertia (i.e. δ=0) that starts from an initial state with no party-dependency ($\theta^{B}(0)=\theta^{R}(0)$). The case δ=0 describes a highly reactive population where individuals choices are driven by the direction of the net effect of in-group neighbors and out-group animosity and not the amount. Then, we extend the results to more general settings with homophily, and party-dependent initial states ($\theta^{B}(0)\neq \theta^{R}(0)$).

### Emergence of polarization in a fully connected network

Consider the case where choice-1 is initially equally popular in both groups ($\theta^{B}(0)=\theta^{R}(0)$). This describes the early COVID-19 pandemic, when Democrats and Republicans were equally cautious about the disease and chose to mask. Remarkably, the long-term outcomes that emerge from a symmetric initial state can be characterized by just two quantities: the ratio of in-group love to out-group hate α/β and the ratio of group sizes r/(1−r).

Theorem 1 **(Information cascades in a fully connected network with affective polarization)**

Consider Eq. 4 which represents the dynamics of the state of the population $\theta (t)=[\theta^{B}(t),\theta^{R}(t){]}^{\prime }$ under the proposed model in a fully connected graph. Let δ=0 (i.e. no inertia) and $\theta^{B}(0)=\theta^{R}(0)$ (i.e. initial state is party independent). Then, the following statements characterize the asymptotic state of the system for various different values of *α* (level of in-group love), *β* (level of out-group hate), and *r* (fraction of red nodes in the network):

- Case 1: Let $\frac{\beta }{\alpha }<\frac{r}{1-r}<\frac{\alpha }{\beta }$. If $\theta^{B}(0)=\theta^{R}(0)>0.5$, then $\lim_{t⟶\infty }\theta (t)=[\theta_{*}^{B},\theta_{*}^{R}{]}^{\prime }=[1,1{]}^{\prime }$. If $\theta^{B}(0)=\theta^{R}(0)<0.5$, then $\lim_{t⟶\infty }\theta (t)=[\theta_{*}^{B},\theta_{*}^{R}{]}^{\prime }=[0,0{]}^{\prime }$ i.e. there is no polarization and both groups fully adopt the choice that was initially more popular.
- Case 2: Let $\frac{r}{1-r}>\frac{\alpha }{\beta }$ and $\frac{r}{1-r}>\frac{\beta }{\alpha }$. If $\theta^{B}(0)=\theta^{R}(0)>0.5$, then $\lim_{t⟶\infty }\theta^{R}(t)=[\theta_{*}^{B},\theta_{*}^{R}{]}^{\prime }=[1,0{]}^{\prime }$. If $\theta^{B}(0)=\theta^{R}(0)<0.5$, then $\lim_{t⟶\infty }\theta (t)=[\theta_{*}^{B},\theta_{*}^{R}{]}^{\prime }=[0,1{]}^{\prime }$ i.e. there is party-line polarization and the red group (which is the majority) fully adopt the choice that was initially popular while the blue group fully adopt the other choice.
- Case 3: Let $\frac{r}{1-r}<\frac{\alpha }{\beta }$ and $\frac{r}{1-r}<\frac{\beta }{\alpha }$. If $\theta^{B}(0)=\theta^{R}(0)>0.5$, then $\lim_{t⟶\infty }\theta^{R}(t)=[\theta_{*}^{B},\theta_{*}^{R}{]}^{\prime }=[0,1{]}^{\prime }$. If $\theta^{B}(0)=\theta^{R}(0)<0.5$, then $\lim_{t⟶\infty }\theta (t)=[\theta_{*}^{B},\theta_{*}^{R}{]}^{\prime }=[1,0{]}^{\prime }$ i.e. there is party-line polarization and the blue group (which is the majority) fully adopt the choice that was initially popular while the red group fully adopt the other choice.
- Case 4: Let $\frac{\beta }{\alpha }>\frac{r}{1-r}>\frac{\alpha }{\beta }$. If $\theta^{B}(0)=\theta^{R}(0)$, then $\lim_{t⟶\infty }\theta (t)=[\theta_{*}^{B},\theta_{*}^{R}{]}^{\prime }=[0.5,0.5{]}^{\prime }$. i.e. there is nonpartisan polarization with half of each group adopting choice-1 and the remaining half adopting choice-0.

The limiting states in cases 1–3 (consensus and polarization along party lines) are locally asymptotically stable stationary states of the system in Eq. 4 whereas the limiting state in case 4 is an unstable stationary state of Eq. 4.

**Fig. 1.**
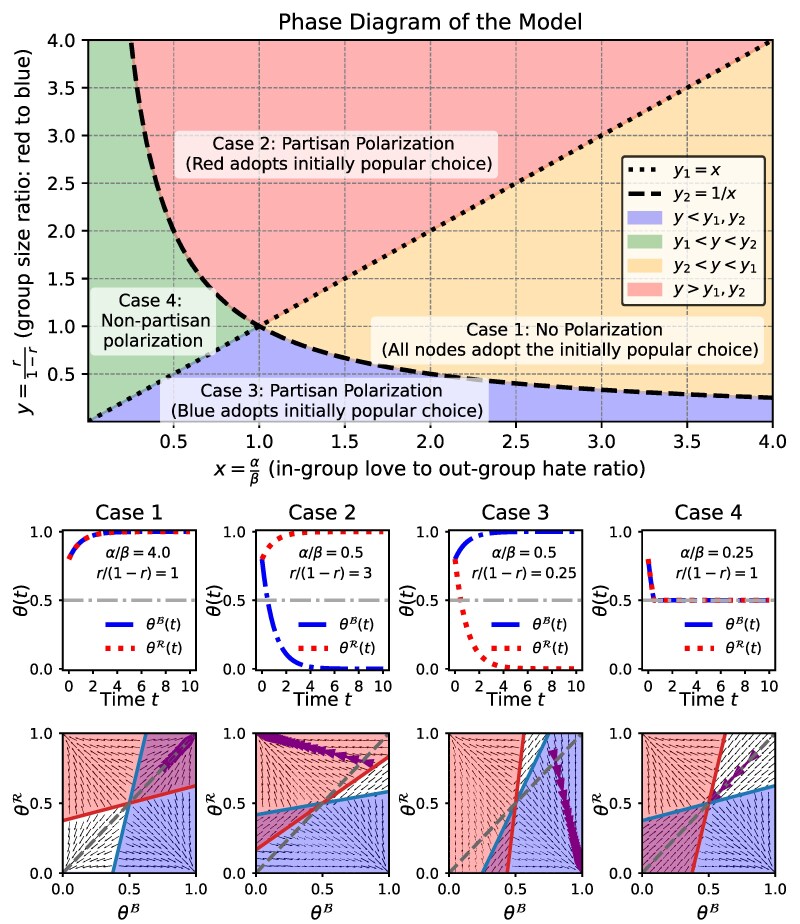
Phase diagram of the model (top) and four example trajectories. The four different regions of the phase diagram (defined by the ratio of in-group love to out-group hate and the ratio of group sizes) lead to different long-term outcomes in a fully connected network when both groups start from the same initial state (i.e. $\theta^{B}(0)=\theta^{R}(0)$). The long-term outcomes are: (case 1, yellow) No Polarization, (case 2, red/case 3, blue) Partisan Polarization, and (case 4, green) Non-Partisan Polarization. Example trajectories in both time-domain and state space are shown below the phase diagram for $\theta^{B}(0)=\theta^{R}(0)=0.8$. The blue and red color areas in state space indicate regions where $\theta^{B}(t),\theta^{R}(t)$ increase (i.e. regions where $p_{\theta }^{B}(0\to 1)=1$ and $p_{\theta }^{R}(0\to 1)=1$ according to Eq. 4). The black arrows in state space plots indicate the path of the differential equation Eq. 4. The purple arrows map the time domain trajectory to the state space.

Figure 1 provides a graphical illustration of the four cases in Theorem 1 containing the phase diagram^e^ (top row) as well as example trajectories in both time domain (second row) and state space (third row). The proof of Theorem 1 is given in Supplementary material, Section B together with additional details (including closed-form expressions of the trajectories of $\theta^{B}(t),\theta^{R}(t)$).

#### Insights from Theorem 1

The four cases in Theorem 1 shed light on the forms of polarization that can emerge in an emotionally divided population starting from a state with no group-level differences: (case 1) global consensus, where all nodes ultimately adopt the same choice, (cases 2 and 3) party-line polarization, where the choices are split along party lines, and (case 4) nonpartisan polarization, where each group is split evenly between the two choices. Below we consider additional insights from Theorem 1.

##### Out-group hate is necessary for polarization:

Note from Fig. 1, that if *β* is approximately zero, then the network will always be in case 1 which achieves consensus from any party-independent initial state $\theta^{B}(0)=\theta^{R}(0)\neq 0.5$.

##### Larger out-group hate relative to in-group love is sufficient for polarization:

When individual choices are driven more by a desire to oppose the out-group than a desire to conform to the in-group, some form of polarization is unavoidable regardless of group sizes. As a result, in the region to the left of the vertical line at α/β=1 in Fig. 1, consensus is not possible. If out-group hate is very high compared to in-group love (α/β≈0 corresponding to case 4), then each group will be evenly split between the two choices. When the disparity between *α* and *β* is not too large compared to group size disparity (i.e. β/α<r/(1−r) or α/β>r/(1−r)), polarization will emerge with the majority adopting the initially more popular choice and the minority adopting the other choice (cases 2 and 3 in Theorem 1). Further, party-line polarization is stable: a small deviation will push the system back to the polarized state as indicated by the arrows pointing to the polarized state in the state space plots of Fig. 1. Additional examples trajectories in the cases where polarization emerge are given in Fig. S4.

##### Larger in-group love relative to out-group hate leads to consensus as long as the group imbalance is not too large:

When the two groups have the same size (i.e. r=0.5), case 1 of Theorem 1 shows that even a slightly larger in-group love compared to the out-group hate (i.e. α>β) is sufficient for the network to adopt the initially popular choice, leading to consensus (see row i of Fig. S5 for an example). Even with unequal group sizes, consensus can be achieved with larger in-group love as long as the group imbalance is not large enough to push the system into case 2 or case 3. In other words, when *α* is sufficiently large compared to *β* , consensus can be achieved even in the presence of unequal group sizes (see row ii of Fig. S5 for an example). Further, note that when *β* is negligible compared to *α*, consensus is always achieved when both groups start from the same initial state (gray diagonal line in state space plots). This highlights our claim that out-group hate is crucial for any form of polarization to occur from a party independent initial state $\theta^{B}(0)=\theta^{R}(0)$. However, even with high in-group love α>β, a large enough group imbalance (r/(1−r)>α/β or r/(1−r)<β/α) can lead to polarization (as shown in row iii of Fig. S5). This observation emphasizes that *more love than hate is necessary but not sufficient for consensus although more hate than love is sufficient for polarization*. In other words, hate is a more powerful divider than love is a unifier in the context of polarization, aligning with the saying that *“bad is stronger than good”* from the psychology literature (31).

##### Majority cannot fully adopt the initially unpopular choice:

When r>0.5 (region above y=1 line in Fig. 1) and $\theta^{B}(0)=\theta^{R}(0)>0.5$ (i.e. choice-1 is initially more popular), there cannot be a case where all of the red group adopts choice-0. In general, starting from a state $\theta^{B}(0)=\theta^{R}(0)$ in a fully connected network, the majority cannot adopt the initially less popular choice.

##### Small perturbations from nonpartisan polarization (case 4) can lead to party-line polarization but not to consensus:

Consider case 4 in Theorem 1 where the population is evenly split between the two choices, regardless of group membership. This stationary state $\theta^{B}(t)=\theta^{R}(t)=0.5$ is unstable, and a small change in $\theta^{B}(t)$ or $\theta^{R}(t)$ can lead the population to polarize along party lines. This can be seen from state space plot corresponding to case 4 in Fig. 1: a small deviation from $\theta^{B}(t)=\theta^{R}(t)=0.5$ caused by a change of either $\theta^{B}(t)$ or $\theta^{R}(t)$ will lead to party-line polarization. For example, if just a few red nodes switch to choice-1 from choice-0, $\theta^{B}(t)$ will converge to 1 and $\theta^{R}(t)$ to 0.

Thus, even on a fully mixed population containing a majority and a minority that are not initially polarized, out-group hate and in-group love alone can lead to the emergence of a wide array of cascading choices.

### Implications for networks with echo chambers

Next, we consider the case where the network G=(V,E) is sampled from a stochastic block model with two communities, where each node is connected to *ρ* fraction of their in-group members and 1−ρ fraction of their out-group members, and *ρ* gives the homophily of the network. Recall from Dynamics of the model on a social network with communities section that the dynamics of the model with homophily can be obtained by replacing *α* and *β* in Eq. 4 with αρ and β(1−ρ), respectively. Consequently, replacing *α* and *β* in Theorem 1 and Fig. 1 with αρ and β(1−ρ) leads to a characterization of the forms of polarization that can emerge in the presence of in-group love, out-group hate, homophily as well as a minority/majority division of the population. This is illustrated in Fig. S1. We now discuss some insights on how these factors can collectively affect the emergence of polarization.

####  

##### Neutral homophily is indistinguishable from the fully connected graph:

When people are neither homophilic nor heterophilic (ρ=0.5), the continuous-time trajectory in a stochastic block model is the same as the continuous-time trajectory in a fully connected graph given in Eq. 4 (since both sides of the inequalities inside indicator functions in Eq. 4 would be multiplied by 0.5). Thus, Theorem 1 as well as insights discussed in Emergence of polarization in a fully connected network section are applicable not only to fully connected graphs but also to Erdos–Rényi random graphs where edges are formed in an independent and identically distributed manner.

##### Highlighting the choices of the out-group in social networks may lead to polarization:

**Fig. 2.**
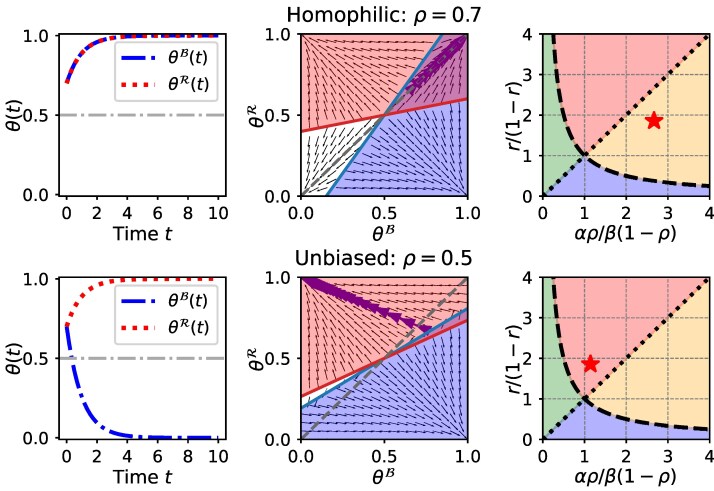
An illustration of how decreasing homophily can cause a party-line polarization. Both figures correspond to α=0.8,β=0.7 (larger in-group favoritism compared to out-group animosity) and r=0.65 (a majority red group). First row corresponds to a homophilic network (intergroup links are more likely to form than intragroup links) with ρ=0.7 whereas second row corresponds to an unbiased network (all links are equally likely to form). Note that decreasing *ρ* from 0.7 (homophily) to 0.5 (unbiased) increases the effect of out-group hate and decreases the effect of in-group love on the choices, and pushes the social network from case 1 (consensus) to case 3 (party-line polarization) in Fig. 1 (with x-axis re-scaled as $\frac{\alpha \rho }{\beta (1-\rho )}$).

A typical approach to reducing partisan divisions calls for increasing the number of cross-party links. For example, consider the case where the two parties are approximately equal in size (r≈0.5) and α>β, which corresponds to case 1 of Fig. 1 where $\frac{\beta }{\alpha }<\frac{r}{1-r}<\frac{\alpha }{\beta }$. Thus, when an individual looks at the entire population (i.e. a fully connected graph) or an unbiased sample of the population (i.e. an Erdos–Rényi random graph), universal consensus is achieved. Then, consider the case where the individual observes others in a biased manner, where each in-group member is observed with probability *ρ* and each out-group member with probability 1−ρ. If ρ<0.5, the out-group will be over-represented compared to its size, amplifying the effect of out-group hate while reducing the effect of in-group love. Thus, the population could move to the red (case 2) or blue regions (case 3) of Fig. 1 where $\frac{\alpha \rho }{\beta (1-\rho )},\frac{\beta (1-\rho )}{\alpha \rho }>\frac{r}{1-r}$ or $\frac{\alpha \rho }{\beta (1-\rho )},\frac{\beta (1-\rho )}{\alpha \rho }<\frac{r}{1-r}$ i.e. partisan polarization can emerge starting from a uniform initial state where the choice is equally popular in both groups. Even a small increase in the number of cross-party links is likely to give rise to polarization (case 2 or case 3) from a nonpolarized state (case 1) when $\frac{\alpha \rho }{\beta (1-\rho )}\approx \frac{r}{1-r}$ or $\frac{\beta (1-\rho )}{\alpha \rho }\approx \frac{r}{1-r}$ (i.e. near the boundaries of case 1 in the phase diagram of Fig. 1 with x-axis re-scaled as $\frac{\alpha \rho }{\beta (1-\rho )}$). Thus, *merely increasing the number of cross-party connections among the two groups may in fact facilitate polarization instead of consensus by amplifying the effect of out-group hate*. Figure 2 shows two different trajectories of θ(t) where the two groups start from the same initial state. Consensus is achieved for a homophilic network (ρ=0.7), where individuals get more information about the in-group, while polarization emerges in an unbiased network (ρ=0.5). This is because decreasing *ρ* from 0.7 to 0.5, pushes the network to case 2 in Fig. 1 (with x-axis re-scaled as $\frac{\alpha \rho }{\beta (1-\rho )}$).

In fact, increased exposure to the out-group (i.e. decreasing *ρ*) can bring divisions to a society already at global consensus. See Fig. S6 for an example. Note that global consensus remains at higher homophily (Case 1 in Fig. 1), and decreasing *ρ* to 0.5 makes the network unbiased but amplifies out-group hate, pushing it to case 3, where the majority stays in the initial state but the minority adopts the choice that no one had chosen at the beginning. Further decreasing homophily makes the network highly heterophilic, where both groups focus largely on the out-group, pushing it to case 4. As this state is unstable, a small deviation causes polarization with one group adopting choice-1 and the other adopting choice-0. Thus, in a society with multiple ideologies, choices being driven by what the *“opposition does”* more than what *“our own group does”* can lead to divisive (Case 2 and Case 3 in Fig. 1) and even unpredictable (case 4 in Fig. 1) polarization of choices, even if the society was initially united. In practice, such situations occur when partisan information sources (e.g. news organizations) emphasize the choices, decisions and actions of the out-group more than those of the in-group.

Relatedly, recall from Eq. 4 that when the two groups are approximately equal in size (i.e. r≈0.5) and ρ=0.5 (unbiased network), people’s choices are driven by $\theta (t)=[\theta^{B}(t),\theta^{R}(t){]}^{\prime }$ i.e. the prevalences of choice-1 in the in-group and out-group. If the popularity of choices is misrepresented in the information they receive at some time instant, that itself could lead to polarization. For example, consider *latte drinking* as the choice and assume that it is equally prevalent among liberals and conservatives. However, if conservatives are selectively exposed to latte-drinking liberals, giving the perception that latte drinking is highly prevalent among them, that may cause them to give up lattes due to the out-group hate effect, and that in turn would lead liberals to further embrace it. Once this divergence takes off, it will be further amplified by the in-group love, leading to the eventual polarization of a seemingly nonpartisan choice (1). Thus, even if a choice is not initially polarized, making it appear to be so in the news or on social media by selectively emphasizing the out-group, can eventually lead to polarization in the form of a self-fulfilling prophecy. This serves as one possible explanation of why even traits that are historically nonpartisan, such as the preferred choice of beverage, leisure activity, vocabulary, etc., can start to diverge along party lines when the prevalence of that trait in the opposite party is emphasized in the digital news (13). Hence, news and social media platforms should take steps to avoid giving the perception of a choice being a partisan signal (e.g. via content and link recommendation algorithms) in order to avoid them actually ending up being partisan issues.

### Group-dependent initial states

When choices are not initially identically distributed in the two groups, several interesting phenomena can emerge. The differential equation in Eq. 4 (and its generalization to stochastic block models) can be used to study such phenomena as well. We begin by stating a result which characterizes conditions that lead to consensus from a party-dependent initial state.

Theorem 2 **(Consensus from party-dependent initial states)**

Consider dynamics of the model on a fully connected graph given in Eq. 4 with δ=0 (i.e. no inertia). Consensus emerges from a group-dependent initial state $\theta^{B}(0)\neq \theta^{R}(0)$ if and only if,

1. $\frac{\beta }{\alpha }<\frac{r}{1-r}<\frac{\alpha }{\beta }$, and,
2. the initial state satisfies $\frac{\beta }{\alpha (1-r)}<\frac{2\theta^{B}(0)-1}{2\theta^{R}(0)-1}<\frac{\alpha }{\beta (1-r)}$.

The first condition of Theorem 2 states that the system has to be in case 1 of Fig. 1, which ensures that consensus is a stable steady state of the system. The second condition of Theorem 2 states that initial distribution of the choices within the groups cannot be too different from each other. The two conditions collectively ensure that consensus is reachable from the initial state. Any parameter configuration (α,β,r) or an initial state that does not satisfy the two conditions will give rise to polarization. The result further highlights the difficulties that lie in the path towards consensus in an affectively polarized society: even with high in-group love and balanced group sizes, the initial differences between the two parties can lead to polarized choices. In order to avoid this, social and news media through which people estimate the choice distributions must avoid emphasizing the differences between groups of different political ideologies.

####  

##### A group can flip:

**Fig. 3.**
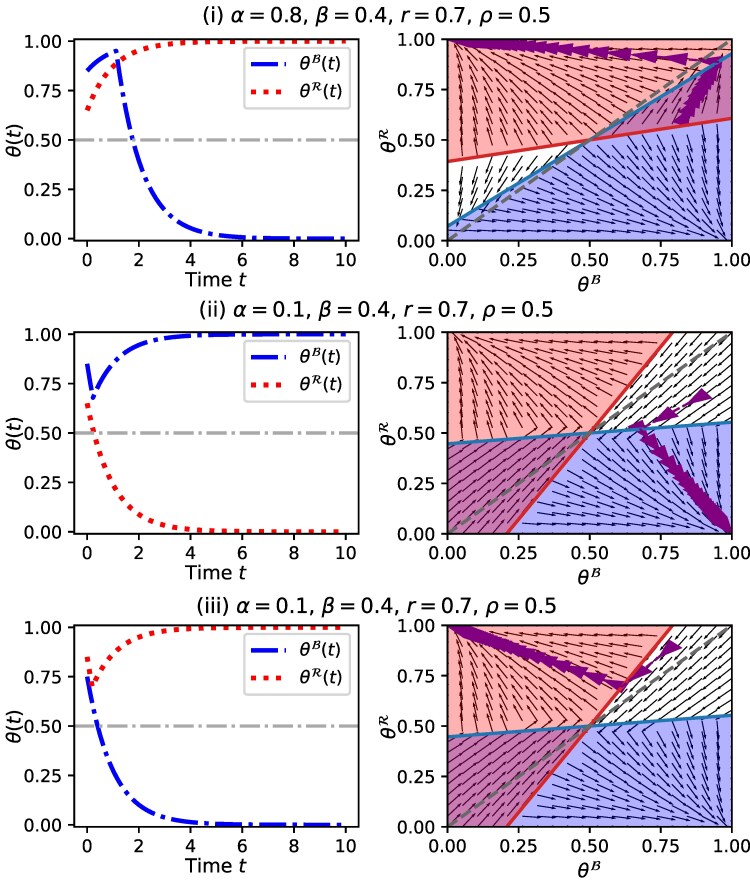
An illustration of three cases where the two groups start at different initial states i.e. $\theta^{B}(0)\neq \theta^{R}(0)$, and one group reverses its direction. In cases i and ii, the minority blue group reverses its direction. In case iii, the majority red group reverses its direction. The blue and red lines in state space indicate the *tipping points* in opinion landscape where the respective group reverses its trend when the trajectory reaches it. The proposed model can demonstrate a variety of such phenomena when the initial states are different for the two groups.

When the groups start from different initial states, their trajectories can change direction. For example, consider the three cases in Fig. 3. In case i of Fig. 3, in-group love is higher than out-group hate (i.e. α>β) and choice-1 is initially more prevalent within each group but to a different degree. Due to higher in-group love, each group initially begins to embrace the choice-1 that is more popular within it. However, as this choice becomes more popular in the majority red group, the opposition intensifies in the minority blue group, which starts to adopt choice-0, leading to the eventual polarization. Interestingly, the *flip* occurs when the population is very closer to consensus. This represents how political negotiations in an affectively polarized society can very unexpectedly break down even when they are on the verge of reaching bi-partisan agreements: the high presence of the same choice in both groups amplifies the effect of out-group hate. More precisely, in-group love is high enough to get closer to consensus (due to the satisfied second condition of Theorem 2), but it is not high enough to make consensus a stable stationary state (due to violated first condition). More in-group love would drive both groups to consensus by focusing on unity within their own party rather than on hate towards the other party. Cases ii and iii of Fig. 3 show scenarios with higher out-group hate where both conditions of Theorem 2 are violated. In case ii, choice-1 is initially more prevalent in both groups but they both initially start adopting choice-0 due to higher out-group hate. However, as choice-0 becomes the more prevalent among the majority, the minority blue group starts adopting choice-1. Eventually, the trajectories converge in the opposite direction. Case iii of Fig. 3 shows a similar scenario where the majority red group reverses the trend. The theoretical tractability of the model Eq. 4 helps identify the exact trajectories for any initial state as seen from Fig. 3.

##### The majority can eventually fully adopt the initially less popular choice:

Unlike the setting where both groups start in the same initial state, the majority can fully adopt the initially less popular choice when the two groups start in different initial states. Figure S7 shows an example of a case where choice-1 is initially more popular among both groups: $\theta^{B}(0)=0.9$ and $\theta^{R}(0)=0.6$. Also, 60% of the nodes in the network are red, making it the majority. However, the red group eventually abandons choice-1 due to the out-group hate effect resulting from the high popularity of choice-1 among the blue group (despite a smaller *β*). In other words, due to high initial unity of the minority blue group, the majority red group is driven more by a desire to oppose the blue party than to unite within their party. The minority blue group fully adopts choice-1 due to the higher in-group love effect created collectively by larger *α* and the high initial popularity of choice-1 within their group.

## Experiments with real-world networks

In this section, we evaluate the proposed model on two real-world social network datasets from Facebook and Brightkite to illustrate that the dynamics of the model on these networks align closely with the theoretically derived expressions and insights. The Facebook dataset (32) contains 4,039 nodes and 58,228 edges, while the Brightkite dataset (33) contains 88,234 nodes and 214,078 edges. Using these two datasets, we first explore how the insights obtained under the unbiased (i.e. no party homophily, or equivalently, fully connected) assumption agree with dynamics on real-world networks. We then explore the implications of community structure and party homophily.

### Unbiased (nonhomophilic) network setting

For each network, the dynamics are obtained as follows for any model parameter configuration α,β,r and initial states $\theta^{B}(0),\theta^{R}(0)$. First, a random fraction *r* of network nodes are assigned to the red group and the rest to the blue group. This assignment of parties (node colors) independent of everything else leads to neutral homophily (i.e. neither homophilic nor heterophilic). Then, a fraction $\theta^{R}(0)$ of red nodes are initialized with dynamic attribute 1 and the remaining red nodes are initialized with the dynamic attribute 0. The initial dynamic attributes of the blue group are similarly assigned according to $\theta^{R}(0)$. Then, at each time step, a node from the network is chosen uniformly at random and its dynamic attribute is updated according to Eq. 2.

**Fig. 4.**
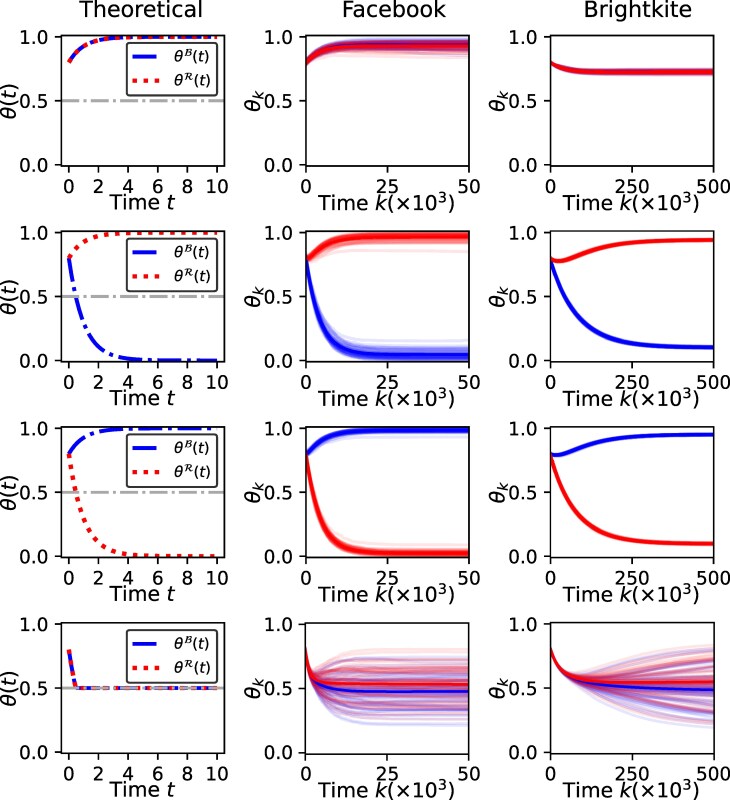
The figure shows the trajectories of the model on an unbiased network (column 1—theoretical trajectories for the stochastic block model outlined in Dynamics of the model on a social network with communities section with ρ=0.5) and two real-world social networks (column 2—Facebook and column 3—Brightkite). Both groups start from the same initial state ($\theta^{B}(0)=\theta^{R}(0)$) and the model parameters (α,β,r) for the four rows correspond to the four cases shown in Fig. 1. It can be seen that the theoretically predicted trajectory (column 1) closely resembles the trajectories for both real-world networks (columns 2 and 3) in each case.

**Fig. 5.**
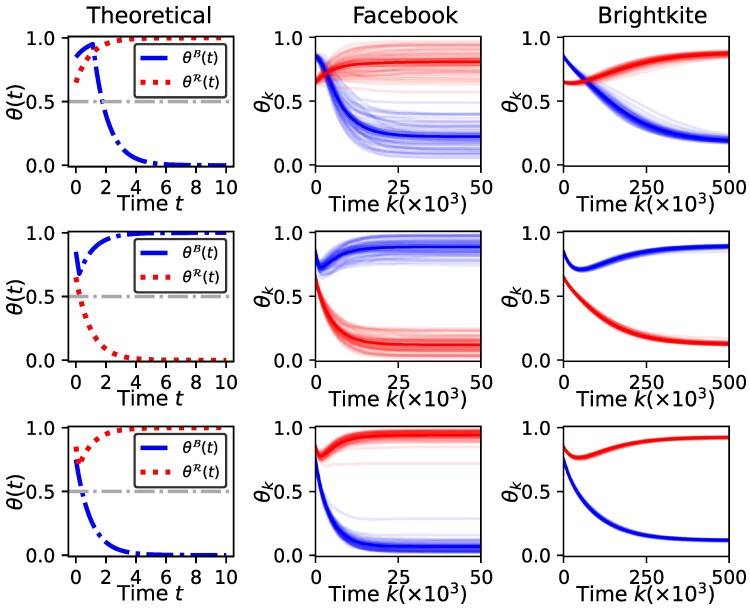
The figure shows the trajectories of the model on an unbiased network (column 1—theoretical trajectories for the stochastic block model outlined in Dynamics of the model on a social network with communities section sec with ρ=0.5) and two real-world social networks (column 2—Facebook and column 3—Brightkite). The groups start from the different initial states ($\theta^{B}(0)\neq \theta^{R}(0)$) and the model parameters (α,β,r) for the three rows correspond to the three cases shown in Fig. 3. It can be seen that the theoretically predicted trajectory (column 1) closely resembles the trajectories for both real-world networks (columns 2,3) in each case.

We consider seven different configurations of the model parameters α,β,r: the results using four different configurations with $\theta^{B}(0)=\theta^{R}(0)$ (same as Fig. 1) are given in Fig. 4 and results with three different configurations with $\theta^{B}(0)\neq \theta^{R}(0)$ (same as Fig. 3) are given in Fig. 5. For each parameter configuration, the first column shows the theoretically derived trajectory $\theta (t)=[\theta^{B}(t),\theta^{R}(t){]}^{\prime }$ under the assumption of an unbiased network with neutral homophily (i.e. the stochastic block model discussed in Dynamics of the model on a social network with communities section with ρ=0.5 that is similar to an Erdos–Rényi graph)^f^. Column 2 (Facebook) and column 3 (Brightkite) show 50 independently simulated trajectories of $\theta_{k}=[\theta_{k}^{B},\theta_{k}^{R}{]}^{\prime },k=0,1,2,\ldots$. The shaded blue and red areas indicate the 95% CI of the trajectories of $\theta_{k}^{B}$ and $\theta_{k}^{R}$, respectively. Several important observations can be made from the results as we discuss next.

####  

##### The dynamics on real-world network resemble the theoretically predicted model dynamics for party-independent initial states:

For each considered parameter configuration in Fig. 4 where $\theta^{B}(0)=\theta^{R}(0)$, the trajectories on both real-world networks closely agree with the theoretically predicted behavior under the unbiased (or equivalently, the fully connected) assumption. In particular, the emergence of consensus (Fig. 4: row 1) and partisan polarization (Fig. 4: rows 2 and 3) can be clearly observed in both Facebook and Brightkite datasets. Further, the unstable nature of the nonpartisan polarization can also be seen in both real-world networks where both groups approach nonpartisan polarization ($\theta^{B}(t)\approx \theta^{R}(t)\approx 0.5$) and then the trajectories show a divergence. This remarkably close agreement with theoretical predictions (under fully connected or unbiased assumptions) indicates the practical usefulness of the model and analysis. Specifically, it illustrates how our theoretical results, such as the phase diagram in Fig. 1 derived under fully connected assumption, are useful to understand dynamics of affective polarization on real-world networks. The empirical results also show how the proposed model can be used in any real-world network to investigate the dynamics of affective polarization.

##### The theoretically predicted and empirically observed dynamics agree for party-dependent initial states as well:

As seen from Fig. 5, the theoretically predicted dynamics align closely with the empirically observed trajectories when initiated from party-dependent initial states ($\theta^{B}(0)\neq \theta^{R}(0)$) as well. In particular, the real-world networks illustrate the emergence of partisan polarization in all three cases of Fig. 5. The cases corresponding to rows 2,3 of Fig. 5 clearly show even the tipping points where one group reverses their trend.

While there is a close alignment between the theoretically predicted dynamics and the dynamics observed via real-world network structures, they are not exactly the same. For example, unlike the fully connected networks, $\theta_{k}^{B},\theta_{k}^{R}$ approach but do not fully converge to 1 or 0 in the real-world networks but become stationary after getting closer to the theoretically predicted value. This deviation is more visible in Brightkite network compared to Facebook (for example, in Fig. 4: row 1). Relatedly, we also note that trajectories on the Facebook network indicate a closer agreement with the theoretical predictions. However, Facebook dynamics also has larger variance (at any given time instant) compared to the Brightkite network. A reason for the larger variance could be the fact that the Facebook network is structurally richer compared to the Brightkite network, with a larger clustering coefficient, a smaller diameter and more closed triangles.

### Implications of homophilic communities

In this section, we focus on homophilic and heterphilic networks (as opposed to the neutral homophilic cases we focused in the previous section). The key aim is to illustrate the validity of the insights from Implications for networks with echo chambers section (about community structure, homophily and heterophily) in real-world network settings.

**Fig. 6.**
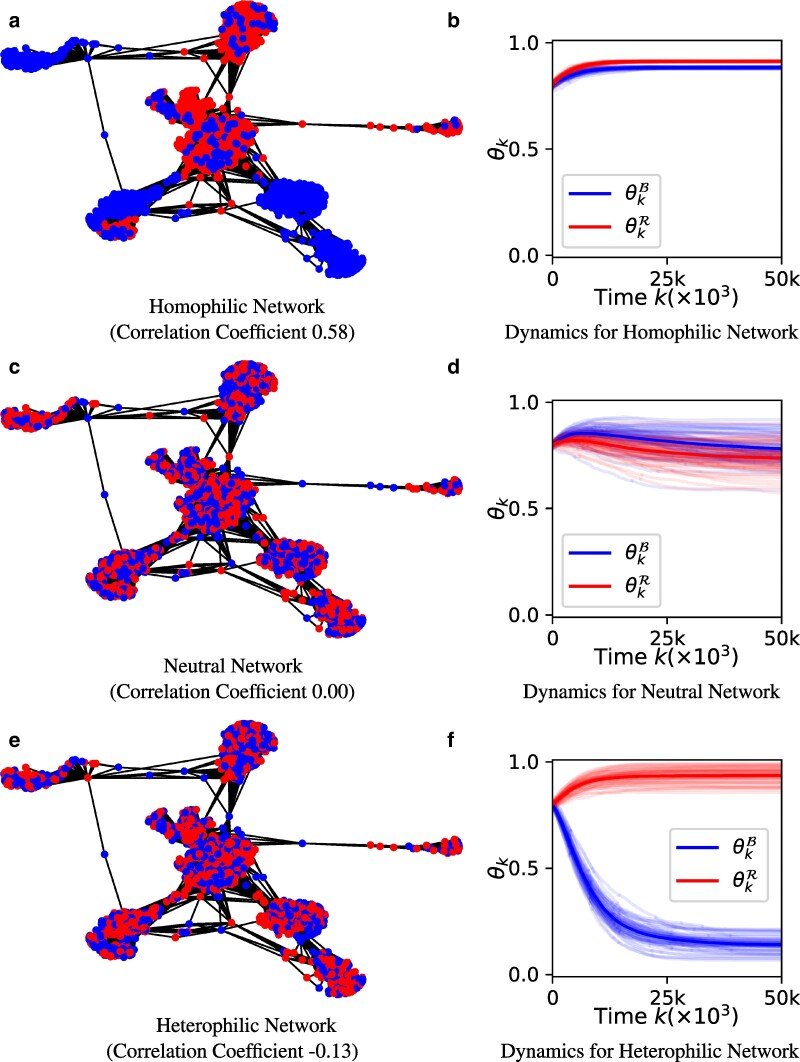
The effect of homophily and heterophily on dynamics of affective polarization illustrated via the Facebook dataset with α=0.7,β=0.5, and r=0.53. The homophilic (a), neutral (c) and heterophilic (e) node color assignments lead to three different behaviors. Compared to the dynamics under the neutral assignment (d), homophily facilitates consensus (b) and heterophily facilitates partisan polarization (f). This empirical result supports our theoretical finding that high exposure to the out-group can amplify party-line polarization.

We utilize The Facebook dataset (32) (described earlier) to explore the implications of homophilic communities as its high average clustering coefficient (0.61) (compared to the Brightkite network: 0.17) helps better study communities and homophily (see Fig. S2 for a visual illustration obtained using Louvain method (34)). We consider three different assignments of the parties (red and blue) to the nodes while keeping the fraction of red nodes r=0.53 fixed for all three assignments (see Supplementary material, Section D.1 for details): Fig. 6(a) homophilic (party assortativity is 0.58), Fig. 6(c) unbiased (party assortativity is 0.00), and Fig. 6(e) heterophilic (party assortativity is −0.13). Then, the model was implemented for each network with initial state $\theta^{B}(0)=\theta^{R}(0)=0.8$ and α=0.7,β=0.5. Each panel on the second column of Fig. 6 shows 50 independently simulated trajectories of $\theta_{k}=[\theta_{k}^{B},\theta_{k}^{R}{]}^{\prime },k=0,1,2,\ldots$ where the shaded blue and red areas indicate the 95% CI of the trajectories of $\theta_{k}^{B}$ and $\theta_{k}^{R}$, respectively.

####  

##### Homophilic communities can facilitate consensus in an affectively polarized society:

It can be seen that homophilic network achieves near perfect consensus with both groups almost fully adopting the dynamic attribute 1 eventually ($\theta_{k}^{B}\approx \theta_{k}^{R}\approx 0.9$ for large *k*). With the exact same parameters, the heterophilic network lead to party-line polarization with the red group largely adopting the dynamic attribute 1 and the blue group adopting the dynamic attribute 0 ($\theta_{k}^{B}\approx 0m,\theta_{k}^{R}\approx 1$ for large *k*). In the neutral case, $\theta_{k}=[\theta_{k}^{B},\theta_{k}^{R}{]}^{\prime }$ does not polarize but also does not fully unite with ∼80% (on average) individuals in each group eventually adopting the dynamic attribute 1. These results support the theoretical results in Implications for networks with echo chambers section. In particular, even though the in-group love α=0.7 exceeds the out-group hate β=0.5, the heterophily amplifies the out-group hate enough to cause party-line polarization. This result highlights why breaking up echo chambers in real-world social networks should be done in a careful manner to avoid facilitating polarization.

Interestingly, we also note that the network in Fig. 6e is only slightly more heterophilic compared to the neutral (unbiased) network in Fig. 6c. Yet, this relatively small heterophily is large enough to cause clearly visible party-line polarization as opposed to the near-consensus achieved in unbiased network. This observation indicates how the proposed model can be useful for understanding the importance of network properties on the dynamics of affective polarization as well as for devising strategies to prevent polarization.

## Conclusion

This article introduced a dynamical model of decision making in a society where people trust the choices of those with same political views while distrusting the choices of those with opposing political views. The model is theoretically tractable and reveals the conditions for the emergence of consensus and partisan divisions from the initial state where there are no divisions. Our analysis highlights the importance of intergroup animosity in driving partisan division. Not only does out-group hate enable party-line polarization, but when it is larger than in-group love, consensus is no longer achievable. In particular, *more hate than love is sufficient for partisan divisions while more love than hate is necessary for consensus*. When partisan mass media emphasize the choices of the out-group more than in-group (i.e. focusing on the other group more than own group), it amplifies the effects of out-group hate and facilitates the emergence of polarization. This may create self-fulfilling prophesies where the perceptions of polarization actually give rise to polarization and explains why, counter to our intuition, cross-party exposure facilitates polarization rather than deterring it. High out-group hate can shatter consensus even when both parties are on the brink of agreement, a trend that is becoming increasingly common within emotionally polarized societies. Further, results obtained by implementing the model on two real-world social network datasets (Facebook and Brightkite) show a close agreement with the theoretical results. The model and its theoretical tractability will also be useful to computational social scientists and network scientists to model the implications of affective polarization in future research and to gain insights on how to avoid its adverse implications on society.

### Limitations and future directions

The proposed model and its analysis has limitations that open up directions for future research. First, our main results assumed a setting with fixed (static) model parameters (α,β). Although we briefly illustrated how the model can be extended to time-varying parameters, a systematic study supported with empirical evidence on how affective polarization (α,β) vary together with the ideological and opinion polarization (θ(t)) is a timely direction of research. Game theoretical (such as (35)) and dynamical system-based methods may be useful in this direction. Also, the model that we proposed assume that people make choices primarily by observing others’ choices instead of the consequences of such choices. Though this approach is suitable for analyzing settings such as the choice of drinks, choice of leisure activities, etc., people do look at the consequences of choices (i.e. whether the result of the choice has been positive or negative in the past) when making more important decisions. Such examples include personal health choices (e.g. vaccines, abortion) and financial decisions. Improving the model to consider how people incorporate consequences of their past choices as well as the choices of their neighbors remains an important direction for future research. Bayesian social learning methods (36) may be useful in this direction. Further, our theoretical analysis of the model was done under simplifying assumptions on the structure of the network (e.g. fully connected networks, Erdos–Rényi type networks, stochastic block models). Extending the insights obtained under those assumptions to further types of network models (e.g. small world model, preferential attachment model, etc.) would provide a better picture of the implications of the network structure on dynamics of affective polarization. Similarly, the dynamics of the network (37) itself can also be incorporated into the model via approaches such as network rewiring (38, 39). Relatedly, exploring how various network properties (e.g. degree distribution, community structure, diameter) as well as network scientific phenomena (e.g. perception bias (40, 41)) can affect the dynamics of affective polarization is an interesting future research direction. Another practically important direction is the estimation of model parameters (in-group love and out-group hate) using data collected from online social networks. Such a principled estimation framework can shed light on the role that each factor (in-group love and out-group hate) plays on opinion polarization. Prior work has shown that network scientific phenomena such as the friendship paradox may be useful for devising such estimation methods (42–45). Lastly, our model and results primarily focused on a two-party system similar to the US political landscape. Generalizing the model to include more than two different political parties will make the model applicable to settings beyond two-party systems.

## Supplementary Material

pgaf082_Supplementary_Data

## Data Availability

All codes are publicly available in the following GitHub repository: https://github.com/ComplexInfo/AP. The Facebook and Brightkite datasets used in this study are available via Stanford Large Network Dataset Collection (46).

## Notes

a This leads to a form of signed network such as those studied in Ref. (14).

b We choose masking as the motivating example as it has been at the center of a recent contentious debate during the COVID pandemic, with empirical evidence showing that people’s choices have been heavily affected by party identity (26). Though we use masking as a motivating example, the broad aim of our work is to study how many seemingly nonpolitical decisions such as choice of drink (e.g. “latte drinking liberals” (1), Bud Light boycott (27)), choice of leisure activities (e.g. “bird-hunting conservatives”), personal health precautions (e.g. masking), etc., end up being polarized along party lines.

c In this article, we assume that the parameters α,β are in the interval (0,1) and remain constant. These assumptions can easily be relaxed to include settings such as out-group ignorance (β=0), out-group love (β<0), as well as time varying parameters (i.e. dynamic model parameters). We illustrate these generalizations in Supplementary material, Section E with example scenarios.

d The piece-wise interpolation of $\theta_{k},k=0,1,2,\ldots$ refers to the continuous time trajectory $\theta^{\frac{1}{N}}(t)=\theta_{k}$ for $t\in \left[\frac{k}{N},\frac{k+1}{N}\right)$ for discrete time k=0,1,2,…

e Figure S3 shows the phase diagram with logarithmic axes. Since each axis of the phase diagram is a ratio of values, the logarithmic axes convey symmetry of the role of parameters.

f Recall from Implications for networks with echo chambers section that Erdos–Rényi type networks lead to the same dynamics as fully connected networks. Thus, the theoretically derived trajectories in column 1 of Figs. 4 and 5 can be viewed as based on fully connected networks as well.

## Preprints

An earlier version of this manuscript was posted on Arxiv: https://arxiv.org/abs/2403.16940.
