# Supplementary material for: How out-group animosity can shape partisan divisions: A model of affective polarization
Source: PNAS Nexus. 2025 Mar 11;4(3):pgaf082. doi: 10.1093/pnasnexus/pgaf082 (PMC11927084; doi:10.1093/pnasnexus/pgaf082)
Supplement: pgaf082_Supplementary_Data [file pgaf082_supplementary_data.pdf]

Supplementary Information

A Proof of Convergence and Uniqueness

A.1 Outline of the Proof and Preliminaries

High-level idea of the proof: The proof relies on the fact that the dynamics of $\theta_k, k = 0, 1, \ldots$ are Markovian and the expected value of the next state given the previous state $\mathbb{E}_k \left\{\theta_{k+1}\right\} = \mathbb{E}\{\theta_{k+1} | \theta_k\}, k = 0, 1, \ldots$ can be written as,

$$\begin{bmatrix} \mathbb{E}_k \left\{\theta^{\mathcal{B}}_{k+1}\right\} \\ \mathbb{E}_k \left\{\theta^{\mathcal{R}}_{k+1}\right\} \end{bmatrix} = \begin{bmatrix} \theta^{\mathcal{B}}_k \\ \theta^{\mathcal{R}}_k \end{bmatrix} + \frac{1}{N} \times \begin{bmatrix} \left(1 - \theta^{\mathcal{B}}_k\right) p^{\mathcal{B}}_\theta(0 \to 1) - \theta^{\mathcal{B}}_k p^{\mathcal{B}}_\theta(1 \to 0) \\ \left(1 - \theta^{\mathcal{R}}_k\right) p^{\mathcal{R}}_\theta(0 \to 1) - \theta^{\mathcal{R}}_k p^{\mathcal{R}}_\theta(1 \to 0) \end{bmatrix}, \tag{E1}$$

where $p^{\mathcal{B}}_\theta(0 \to 1), p^{\mathcal{B}}_\theta(1 \to 0), p^{\mathcal{R}}_\theta(0 \to 1), p^{\mathcal{R}}_\theta(1 \to 0)$ were defined in Eq. 4. Therefore, the Markovian dynamics of the proposed model can be expressed as,

$$\theta_{k+1} = \theta_k + \frac{1}{N}(g(\theta_k) + M_{k+1}) \tag{E2}$$

where

$$g(\theta_k) = \begin{bmatrix} g^{\mathcal{B}}(\theta_k) \\ g^{\mathcal{R}}(\theta_k) \end{bmatrix} = \begin{bmatrix} \left(1 - \theta^{\mathcal{B}}_k\right) p^{\mathcal{B}}_\theta(0 \to 1) - \theta^{\mathcal{B}}_k p^{\mathcal{B}}_\theta(1 \to 0) \\ \left(1 - \theta^{\mathcal{R}}_k\right) p^{\mathcal{R}}_\theta(0 \to 1) - \theta^{\mathcal{R}}_k p^{\mathcal{R}}_\theta(1 \to 0) \end{bmatrix},$$

and M_k is a martingale difference noise sequence. Eq. E2 can be viewed as a stochastic approximation with constant step size $1/N$. Thus, for large N, the discrete time trajectory $\theta_k, k = 0, 1, 2, \ldots$ evolves without jumps and it converges to the trajectory of the limit mean differential in Eq. 4. For such constant step-size stochastic approximation algorithms, typical proof approach is to invoke a form of law of large-numbers and establish that the interpolated trajectory of Eq. E2 converges weakly (in distribution) to a differential equation of the form $\dot{\theta}(t) = g(\theta(t))$ as the step size $1/N$ tends to 0. However, since $g(\cdot)$ is a discontinuous function, this typical approach that establishes (weak) convergence to an ordinary differential equation does not work. We establish the weak convergence of the interpolated stochastic

trajectory of the model to a (deterministic) differential inclusion of the form $\dot{x}(t) \in h(x(t))$ where $h(\cdot)$ is a set-valued map constructed using the discontinuous $g(\cdot)$. Any trajectory of the form $x(t) = x(0) + \int_0^t y(s)ds$ satisfying $y(t) \in h(x(t))$ for all t is called a solution to the differential inclusion $\dot{x}(t) \in h(x(t))$. Such solutions are called Filippov solutions to the discontinuous dynamical system $\dot{\theta}(t) = g(\theta(t))$ (or Caratheodory solution of the differential inclusion $\dot{x}(t) \in h(x(t))$)[7] We then show that due to the piece-wise continuous form of $g(\theta_k)$, the solution is unique in all cases Except Case 4 of Theorem 1.

Required results from literature: The proof relies on two results from literature related to discontinuous dynamical systems that we state below. Let the distance between a continuous trajectory $z(\cdot)$ and the solution set $\mathcal{S}_T$ of a differential inclusion $\dot{x}(t) \in h(x(t))$ be defined as,

$$l(z(\cdot), \mathcal{S}_T) \stackrel{\text{def}}{=} \inf_{y(\cdot)\in\mathcal{S}_T} \sup_{t\in[0,T]} ||z(t) - y(t)||. \tag{E3}$$

The following result from (28) is used to establish the weak-convergence of the sample paths.

Lemma 3 ((28)[Adapted from Theorem 9.4). *] Consider the stochastic approximation,*

$$x_{k+1} = x_k + a\left(g(x_k) + M_{k+1}\right), k \geq 0 \tag{E4}$$

where $g(\cdot)$ is measurable and satisfies $||g(x)|| \leq C(1 + ||x||)$ for some $C > 0$. Let

$$h(x) = \bigcap_{\epsilon>0} \bar{co}\left(g(y) : ||y - x|| < \epsilon\right). \tag{E5}$$

where $\bar{co}$ denotes the convex closure. Then,

$$l(x^a(\cdot)|_{[t',t'+T]}, \mathcal{S}_T) \xrightarrow{a\downarrow 0} 0 \tag{E6}$$

uniformly in t' where $x^a(t)$ is the interpolated trajectory of the stochastic approximation algorithm and $\mathcal{S}_T$ is the solution set of the differential inclusion

$$\dot{x}(t) \in h(x(t)). \tag{E7}$$

[7] See (29) for a detailed introduction to discontinuous dynamical systems and their solution concepts.

We will also use (29)[Proposition 5] to establish the uniqueness of the solutions to the differential inclusion. At a high-level, (29)[Proposition 5] states that the Filippov solution of a piece-wise continuous differential equation with a discontinuous right-hand side (i.e., the solutions to the differential inclusion constructed using that differential equation as in E5) is unique if the trajectories that approach the boundary of a continuous region either slides along the boundary or cross into the next region.

A.2 Proof of Convergence

Consider the model proposed in Sec. 2. Note that the value of $\theta^{\mathcal{B}}_{k+1} - \theta^{\mathcal{B}}_{k}$ can take three different values under three events:

Event 1: $\theta^{\mathcal{B}}_{k+1} - \theta^{\mathcal{B}}_{k} = \frac{1}{N^{\mathcal{B}}}$ in the event that X_{k+1} is a blue node that takes action-0 at time k and switches to action-1 at time $k+1$

Event 2: $\theta^{\mathcal{B}}_{k+1} - \theta^{\mathcal{B}}_{k} = -\frac{1}{N^{\mathcal{B}}}$ in the event that X_{k+1} is a blue node that takes action-1 at time k and switches to action-0 at time $k+1$

Event 3: $\theta^{\mathcal{B}}_{k+1} - \theta^{\mathcal{B}}_{k} = 0$ in any event other than Event 1 and Event 3.

Let $\mathbb{P}_k\{\cdot\}, \mathbb{E}_k\{\cdot\}$ denote the probability measure and expected value conditional on all events that have occurred till time k. Consider the Event 1 first. Note that the probability that X_k is a blue node with choice-0 at time k is $\mathbb{P}_k\left\{R(X_{k+1}) = 0 \wedge H_k(X_{k+1}) = 0\right\} = \frac{N^{\mathcal{B}}(1-\theta^{\mathcal{B}}_k)}{N}$. For a fully connected graph, note that the probability that a random blue node with choice-0 at time k switches to the action choice-1 at time $k+1$ can be written as:

$$\mathbb{P}_k\left\{H_{k+1}(X_{k+1}) = 1 | R(X_{k+1}) = 0 \wedge H_k(X_{k+1}) = 0\right\} \tag{E8}$$

$$= \mathbb{P}_k\left\{\alpha\left(d^{in,0}_k(X_k) - d^{in,1}_k(X_k)\right) - \beta\left(d^{out,0}_k(X_k) - d^{out,1}_k(X_k)\right) > 0 | R(X_k) = 0 \wedge H_k(X_k) = 0\right\} \tag{E9}$$

$$= \mathbb{1}\left(\alpha(1-r)\left(2\theta^{\mathcal{B}}_k - 1\right) - \beta r\left(2\theta^{\mathcal{R}}_k - 1\right) > 0\right) \tag{E10}$$

$$= p^{\mathcal{B}}_{\theta}(0 \to 1) \tag{E11}$$

Similarly, we also obtain,

$$p_\theta^{\mathcal{B}}(1 \to 0) = \mathbb{1}\left(\alpha(1-r)\left(2\theta_k^{\mathcal{B}}-1\right) - \beta r\left(2\theta_k^{\mathcal{R}}-1\right) < 0\right). \tag{E12}$$

Therefore, conditional on all events that have occurred till time k, the expected value of $\theta_{k+1}^{\mathcal{B}}$ can be written as:

$$\mathbb{E}_k\left\{\theta_{k+1}^{\mathcal{B}}\right\} = \theta_k^{\mathcal{B}} + \frac{1}{N^{\mathcal{B}}} \times \frac{N^{\mathcal{B}}\left(1-\theta_k^{\mathcal{B}}\right)}{N} \times p_\theta^{\mathcal{B}}(0 \to 1) - \frac{1}{N^{\mathcal{B}}} \times \frac{N^{\mathcal{B}}\theta_k^{\mathcal{B}}}{N} \times p_\theta^{\mathcal{B}}(1 \to 0) \tag{E13}$$

Following similar arguments for the red-group yields similar expressions for $\mathbb{E}_k\left\{\theta_{k+1}^{\mathcal{R}}\right\}$, which yields

$$\begin{bmatrix}\mathbb{E}_k\left\{\theta_{k+1}^{\mathcal{B}}\right\} \\ \mathbb{E}_k\left\{\theta_{k+1}^{\mathcal{R}}\right\}\end{bmatrix} = \begin{bmatrix}\theta_k^{\mathcal{B}} \\ \theta_k^{\mathcal{R}}\end{bmatrix} + \frac{1}{N} \times \begin{bmatrix}\left(1-\theta_k^{\mathcal{B}}\right)p_\theta^{\mathcal{B}}(0 \to 1) - \theta_k^{\mathcal{B}} p_\theta^{\mathcal{B}}(1 \to 0) \\ \left(1-\theta_k^{\mathcal{R}}\right)p_\theta^{\mathcal{R}}(0 \to 1) - \theta_k^{\mathcal{R}} p_\theta^{\mathcal{R}}(1 \to 0)\end{bmatrix}, \tag{E14}$$

where,

$$p_\theta^{\mathcal{B}}(0 \to 1) = \mathbb{1}\left(\alpha(1-r)\left(2\theta^{\mathcal{B}}-1\right) - \beta r\left(2\theta^{\mathcal{R}}-1\right) > 0\right), \quad p_\theta^{\mathcal{B}}(1 \to 0) = 1 - p_\theta^{\mathcal{B}}(0 \to 1)$$
$$p_\theta^{\mathcal{R}}(0 \to 1) = \mathbb{1}\left(\alpha r\left(2\theta^{\mathcal{R}}-1\right) - \beta(1-r)\left(2\theta^{\mathcal{B}}-1\right) > 0\right), \quad p_\theta^{\mathcal{R}}(1 \to 0) = 1 - p_\theta^{\mathcal{R}}(0 \to 1).$$

Thus, the evolution of the state can be expressed as Eq. (E2). Note $g(\cdot)$ in Eq. (E2) satisfies the linear growth condition since it is a piece-wise linear function taking values in $[-1,1]^2$. The set of discontinuities are defined by the states $\theta(t) = [\theta^{\mathcal{B}}(t), \theta^{\mathcal{R}}(t)]'$ that satisfy

$$\alpha(1-r)\left(2\theta^{\mathcal{B}}-1\right) - \beta r\left(2\theta^{\mathcal{R}}-1\right) = 0 \tag{E15}$$

or

$$\alpha r\left(2\theta^{\mathcal{R}}-1\right) - \beta(1-r)\left(2\theta^{\mathcal{B}}-1\right) = 0. \tag{E16}$$

Thus, Lemma 3 implies that the stochastic trajectory of the proposed model converges to the solution set of the differential inclusion

$$\dot{\theta}(t) \in h(\theta(t)) = [h^{\mathcal{B}}(\theta(t)), h^{\mathcal{R}}(\theta(t))]' \tag{E17}$$

where

$$h^{\mathcal{B}}(\theta(t)) = \begin{cases} [-\theta^{\mathcal{B}}(t), 1-\theta^{\mathcal{B}}(t)] & \text{if } \alpha(1-r)\left(2\theta^{\mathcal{B}}-1\right) - \beta r\left(2\theta^{\mathcal{R}}-1\right) = 0 \\ \{g^{\mathcal{B}}(\theta)\} & \text{otherwise,} \end{cases} \tag{E18}$$

$$h^{\mathcal{R}}(\theta(t)) = \begin{cases} [-\theta^{\mathcal{R}}(t), 1-\theta^{\mathcal{R}}(t)] & \text{if } \alpha r\left(2\theta^{\mathcal{R}}-1\right) - \beta(1-r)\left(2\theta^{\mathcal{B}}-1\right) = 0 \\ \{g^{\mathcal{R}}(\theta)\} & \text{otherwise,} \end{cases} \tag{E19}$$

which is the Filippov solution set of the discontinuous differential equation Eq. 4.

A.3 Proof of Uniqueness

To establish the uniqueness of the Filippov solution, we note that any solution to the Eq. E17 which approaches a point of discontinuity except $(0.5, 0.5)$ crosses the boundary and move to the next region. The only setting in which a trajectory approaches $(0.5, 0.5)$ is the Case 4 of Theorem 1 with $\theta^{\mathcal{B}}(0) = \theta^{\mathcal{R}}(0)$. Thus, according to (29)[Proposition 5], all trajectories except Case 4 with $\theta^{\mathcal{B}}(0) = \theta^{\mathcal{R}}(0)$ are unique.

Uniqueness of trajectories can also be seen from state space plots for the four cases in Fig. 1 as well. Note that the only form of trajectory that approaches the boundary but does not cross in to the other region is the trajectory starting with $\theta^{\mathcal{B}}(0) = \theta^{\mathcal{R}}(0)$ in Case 4. All other initial states therefore have unique trajectories.

B Proof of Theorem 1

Let $\delta = 0$ and $\theta^{\mathcal{B}}(0) = \theta^{\mathcal{R}}(0) = c$. Note from the dynamical system in Eq. 4 that the state space $[0,1]^2$ can be partitioned into four regions based on whether $p^{\mathcal{B}}_{\theta}(0 \to 1) = 1$ (blue region in state space plot of Fig. 1), $p^{\mathcal{B}}_{\theta}(0 \to 1) = 0$ (non-blue region in state space plot of Fig. 1), $p^{\mathcal{R}}_{\theta}(0 \to 1) = 1$ (red region in state space plot of Fig. 1), $p^{\mathcal{R}}_{\theta}(0 \to 1) = 0$ (non-red region in state space plot of Fig. 1). In the blue region (resp. red region), the $\theta^{\mathcal{B}}(t)$ (resp. $\theta^{\mathcal{R}}(t)$) increases and in the non-blue (resp. non-red) region the $\theta^{\mathcal{B}}(t)$ (resp. $\theta^{\mathcal{R}}(t)$) decreases.

Case 1: When $\frac{\beta}{\alpha} < \frac{r}{1-r} < \frac{\alpha}{\beta}$, both groups are either in increasing region (if $c > 0.5$) or decreasing region (if $c < 0.5$) at time $t = 0$. Note from the state space plot of Fig. 1 (case 1) that if each of the two groups are in the increasing region (intersection of the blue and red regions) or each of the two groups are in the decreasing region (neither red region nor blue region) at time $t = 0$, they continue to be in that same region at any time instant $t > 0$.

Cases 2, 3: Similarly, conditions in Case 2 and Case 3 guarantee that one group is in the increasing region and the other is in the decreasing region at time time $t = 0$. Note from the state space plot of Fig. 1 (cases 2,3) that the trajectory remains in the same region that it starts at time $t = 0$.

Case 4: Under conditions in Case 4, both groups increase ($c < 0.5$) or decrease ($c > 0.5$) till they reach $[0.5, 0.5]'$ where $\dot{\theta}^{\mathcal{R}}(t) = \dot{\theta}^{\mathcal{B}}(t) = 0$.

Closed-form Expressions for Trajectories: Expressions $\theta^{\mathcal{B}}(t), \theta^{\mathcal{R}}(t)$ for the Cases 1-3 in Theorem 1 can easily be derived in closed-form. Without loss of generality, consider the blue-group. Let $\theta^{\mathcal{B}}(0) = \theta^{\mathcal{R}}(0) = c$ be the initial state of each group.

If $\alpha(1-r)(2c-1) - \beta r(2c-1) > 0$ (i.e., the initial state $\theta(0) = \left[\theta^{\mathcal{B}}(0), \theta^{\mathcal{R}}(0)\right]'$ is in the area indicated by blue color in the state space plots of Fig. 1 indicating that $p_{\theta}^{\mathcal{B}}(0 \to 1) = 1$ at time $t = 0$), then the dynamics of $\theta^{\mathcal{B}}(t)$ is given by

$$\dot{\theta}^{\mathcal{B}}(t) = 1 - \theta^{\mathcal{B}}(t)$$

according to Eq. 4. Solving the above differential equation and determining the constants using the initial conditions yields

$$\theta^{\mathcal{B}}(t) = 1 - (1-c)\mathrm{e}^{-t},$$

which converges to 1 asymptotically.

If $\alpha(1-r)(2c-1) - \beta r(2c-1) < 0$) (i.e., the initial state $\theta(0) = \left[\theta^{\mathcal{B}}(0), \theta^{\mathcal{R}}(0)\right]'$ is not in the area indicated by blue color in the state space plots of Fig. 1 indicating that $p_{\theta}^{\mathcal{B}}(1 \to 0) = 1$

at time $t = 0$), then the dynamics of $\theta^{\mathcal{B}}(t)$ is given by

$$\dot{\theta}^{\mathcal{B}}(t) = -\theta^{\mathcal{B}}(t),$$

whose solution is

$$\theta^{\mathcal{B}}(t) = c\mathrm{e}^{-t},$$

which converges to 0 asymptotically.

The trajectory for the red-group $\theta^{\mathcal{R}}(t)$ can be derived by following similar steps: $p_{\theta}^{\mathcal{R}}(1 \to 0) = 1$ at time $t = 0$ corresponds to $\theta^{\mathcal{R}}(t) = 1 - (1-c)\mathrm{e}^{-t}$ and $p_{\theta}^{\mathcal{R}}(0 \to 1) = 1$ at time $t = 0$ corresponds to $\theta^{\mathcal{R}}(t) = c\mathrm{e}^{-t}$.

Stability of Stationary States: The state space plots corresponding to the Cases 1-3 in Fig. 1 show that consensus and partisan polarization are stable steady states (point attractors). More specifically, a small deviation from consensus or partisan polarization will lead the system back that same state. This can be formally verified by looking at the Jacobian as well: for each stationary state corresponding to cases 1-3, the Jacobian is a diagonal matrix with negative diagonal values.

On the other hand, the non-partisan polarization (corresponding to case 4 in Fig. 1) is unstable. As seen from the state space plot corresponding to case 4, a deviation in any direction other than the diagonal gray line will lead the system to partisan polarization.

C Dynamics of the Model on a Network with Communities

When the graph $G = (V, E)$ is a stochastic block model with in-group link probability ρ and out-group link probability of $1 - \rho$, the piece-wise interpolation of the discrete-time trajectory $\theta_k = [\theta_k^{\mathcal{B}}, \theta_k^{\mathcal{R}}]', k = 0, 1, 2, \ldots$ can be approximated using the continuous-time trajectory $\theta(t) = [\theta^{\mathcal{B}}(t), \theta^{\mathcal{R}}(t)]', t \geq 0$ of the following differential equation as the number of nodes in the graph

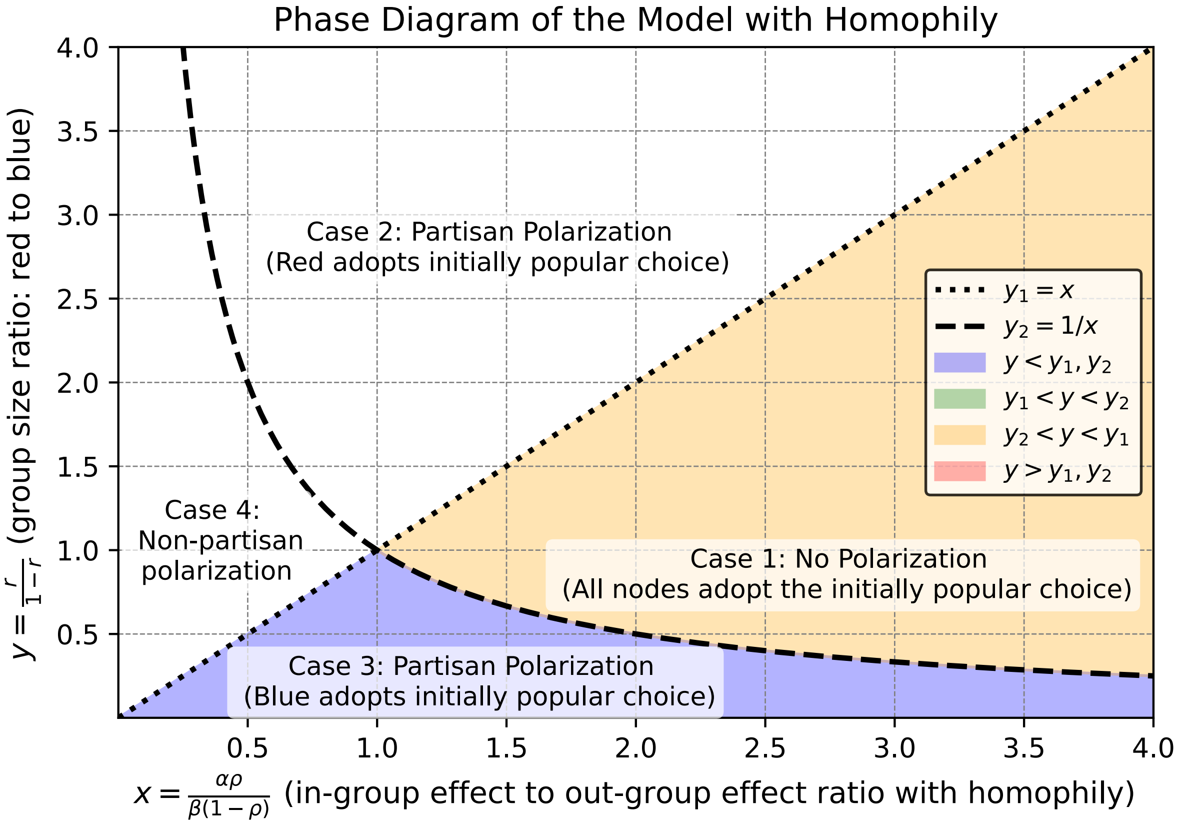

Figure S1: The four different regions of the model parameters (in-group conformity α, out-group dissent β, homophily ρ and fraction of red-nodes r) that lead to different asymptotic behaviors in a stochastic block model type graph starting from an initial state where the distribution of choices is the same for both parties i.e., i.e., $\theta^{\mathcal{B}}(0) = \theta^{\mathcal{R}}(0)$). This figure is similar to the analogous figure for the fully connected graph (Fig. 1) except that the in-group effect is amplified by ρ (probability observing each in-group member) and the out-group effect is amplified by $1 - \rho$ (probability of observing each out-group member).

N is large:

$$\begin{bmatrix} \dot{\theta}^{\mathcal{B}} \\ \dot{\theta}^{\mathcal{R}} \end{bmatrix} = \begin{bmatrix} \left(1-\theta^{\mathcal{B}}\right) p_\theta^{\mathcal{B}}(0 \to 1) - \theta^{\mathcal{B}} p_\theta^{\mathcal{B}}(1 \to 0) \\ \left(1-\theta^{\mathcal{R}}\right) p_\theta^{\mathcal{R}}(0 \to 1) - \theta^{\mathcal{R}} p_\theta^{\mathcal{R}}(1 \to 0) \end{bmatrix}, \tag{E20}$$

where,

$$\begin{aligned}
p_\theta^{\mathcal{B}}(0 \to 1) &= \mathbb{1}\left(\alpha\rho(1-r)\left(2\theta^{\mathcal{B}}-1\right) - \beta(1-\rho)r\left(2\theta^{\mathcal{R}}-1\right) > \delta\right) \\
p_\theta^{\mathcal{B}}(1 \to 0) &= \mathbb{1}\left(\alpha\rho(1-r)\left(2\theta^{\mathcal{B}}-1\right) - \beta(1-\rho)r\left(2\theta^{\mathcal{R}}-1\right) < -\delta\right) \\
p_\theta^{\mathcal{R}}(0 \to 1) &= \mathbb{1}\left(\alpha\rho r\left(2\theta^{\mathcal{R}}-1\right) - \beta(1-\rho)(1-r)\left(2\theta^{\mathcal{B}}-1\right) > \delta\right), \\
p_\theta^{\mathcal{R}}(1 \to 0) &= \mathbb{1}\left(\alpha\rho r\left(2\theta^{\mathcal{R}}-1\right) - \beta(1-\rho)(1-r)\left(2\theta^{\mathcal{B}}-1\right) < -\delta\right)
\end{aligned}$$

Consequently, the analogous version of the Fig. 1 for stochastic block models is shown in Fig. S1.

D Additional Details and Results

D.1 Generating Networks with Different Levels of Homophily

We first partitioned the network into communities using the Louvain method (*34*), resulting in 16 communities and a modularity of 0.83. The resulting network and the communities are shown in Fig. S2. Next, we assign party (color) membership to nodes as follows. For the largest of 16 clusters, we assign 90% of the nodes randomly to be blue and the rest red. For the second largest cluster, we assign 90% of the nodes randomly to be red and the rest to blue.[8] This alternating majority and minority assignment was repeated for all 16 clusters. At the end, we obtained a network with 53% of all nodes in the red group and a party-assortativity coefficient of 0.58 (i.e., homophilic) that is shown in Fig. 6(a).

To obtain a neutral party assignment (that is neither homophilic and nor heterophilic), a random permutation of the above obtained homophilic party assignment was used. This yielded the

[8]The value 90% was used so that it results in approximately a fraction of 50% red nodes at the end with the cluster sizes.

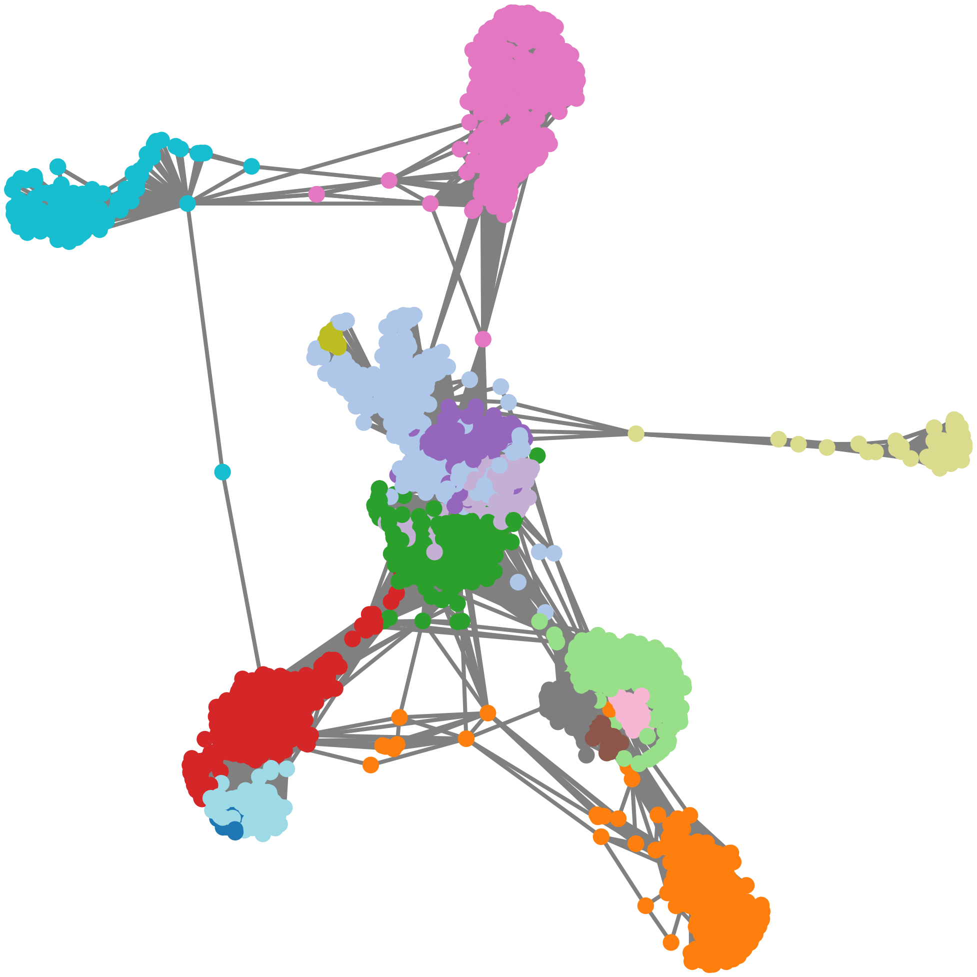

Figure S2: The 16 communities of the Facebook network obtained via the Louvain method that maximizes the modularity. The shown community partitioning has a modularity of 0.83. This commuity structure was exploited to generate the party-homophilic network shown in Fig. 6(a)

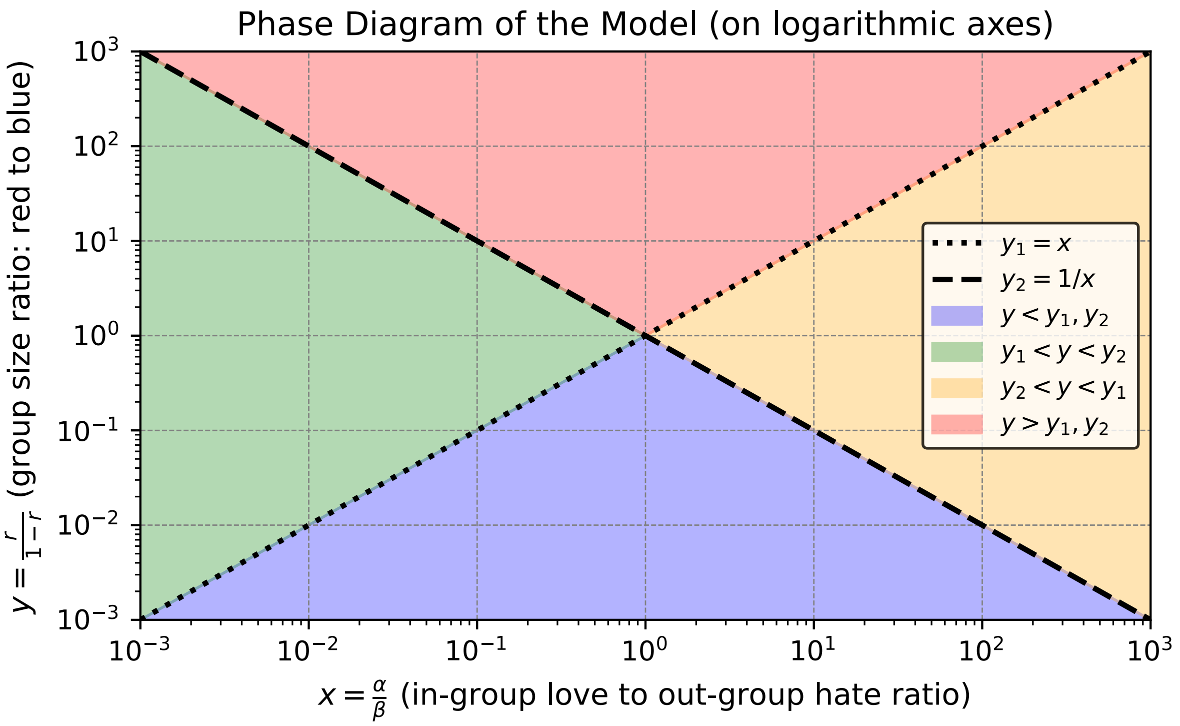

Figure S3: Phase diagram of the model on logarithmic axes. The logarithmic axes better represent the symmetric nature of the four regions.

graph shown in Fig. 6(c) where 53% of all nodes were red and the party-assortativity coefficient is 0.00 (i.e., neutral).

To obtain a heterophilic party assignment, we started with the neutral party assignment from the previous case. Then, two edges $(u1, v1), (u2, v2)$ were randomly chosen. The parties of $u1$ and $u2$ were interchanged and the parties of $v1$ and $v2$ were interchanged, if this action results in a decreased party assortativity. This process was repeated for 100k iterations, resulting in the party assignment shown in Fig. 6(e) that corresponds to a party assortativity coefficient of -0.13 (i.e., heterophilic).

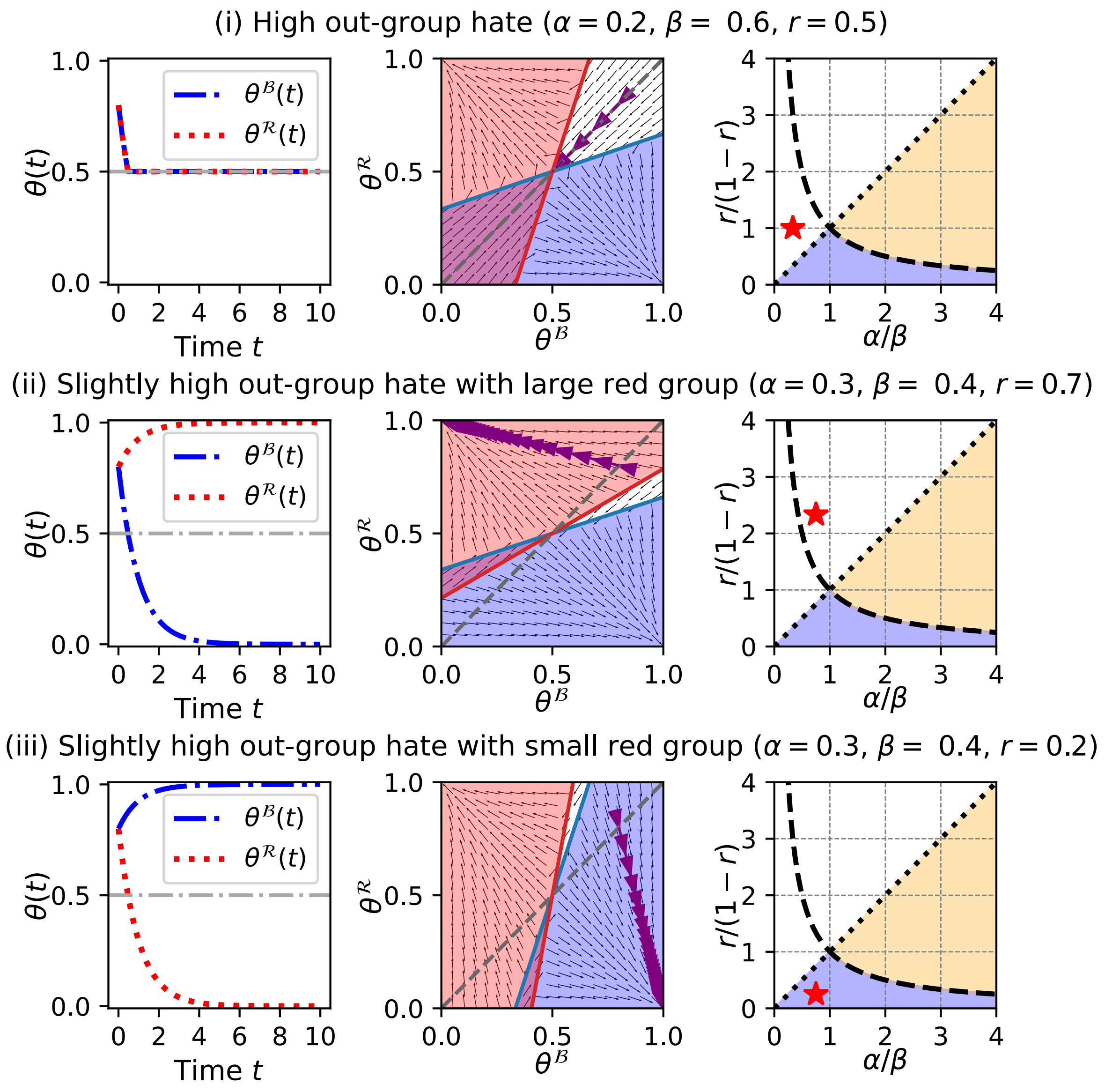

Figure S4: Example trajectories of the state when the out-group hate β is larger than in-group love α. The trajectories $[\theta^{\mathcal{B}}(t), \theta^{\mathcal{R}}(t)]$ over time (left column) and in the state space (middle column) show the evolution of $\theta(t) = [\theta^{\mathcal{B}}(t), \theta^{\mathcal{R}}(t)]$. The blue and red colors in middle column indicate regions where $\theta^{\mathcal{B}}(t), \theta^{\mathcal{R}}(t)$ increase (i.e., regions where $p_{\theta}^{\mathcal{B}}(0 \to 1) = 1$ and $p_{\theta}^{\mathcal{R}}(0 \to 1) = 1$ according to Eq. 4). The black arrows in state space plots (middle column) indicate the path of the differential equation Eq. 4. The yellow arrows corresponds to the time domain trajectory (in left column). The figure shows how either uniform (row i) or party-line polarization (row ii and row-iii) can emerge when people are driven largely by their opposition to the out-group than their adherence to the in-group. Further, uniform polarization that emerges in the presence of very high out-group hate is unstable since some black arrows point away from $[0.5, 0.5]$ as seen from the state space plots (middle column) of row-i. In this case, small deviations from non-partisan polarization can lead to partisan polarization .

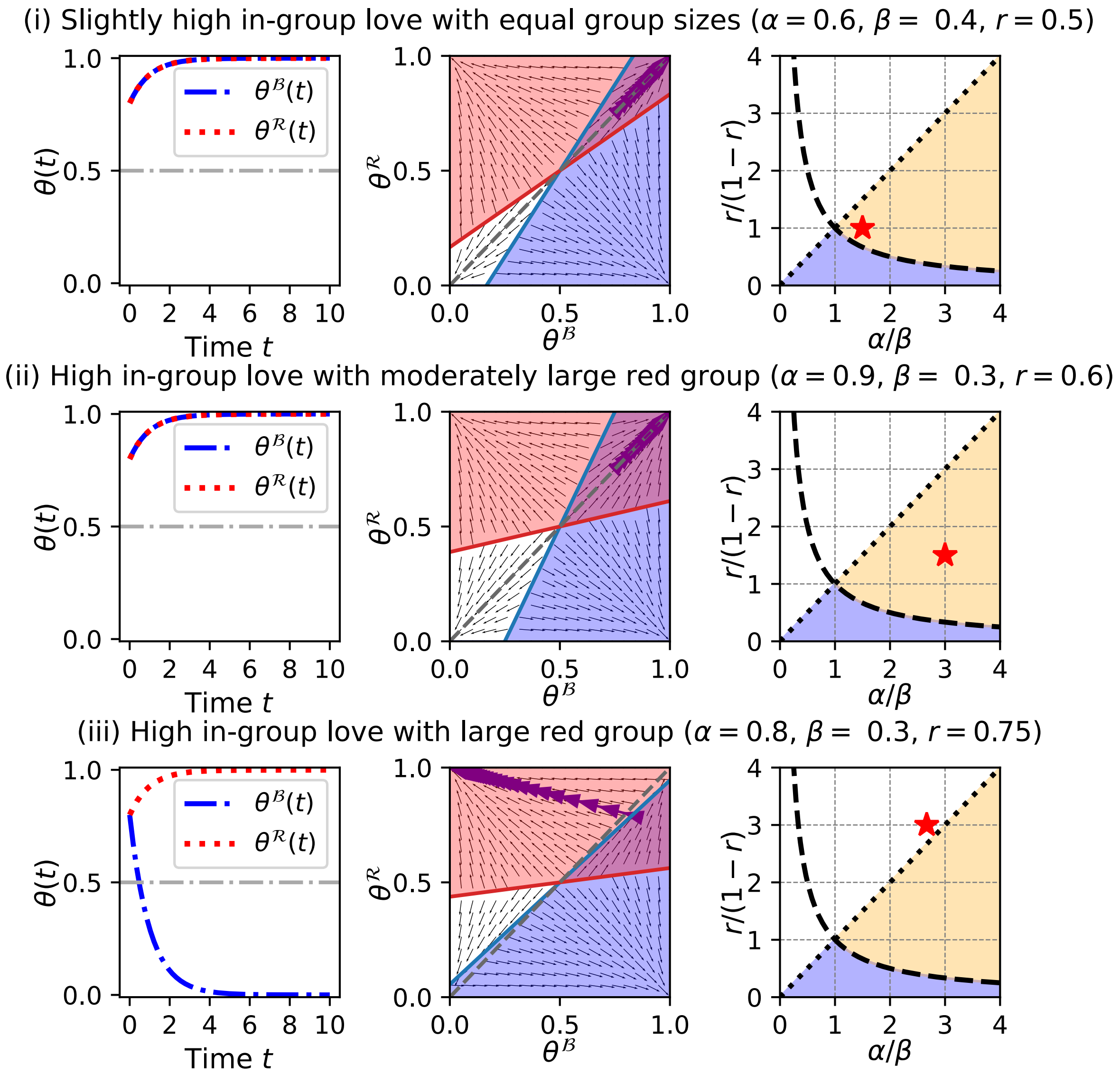

Figure S5: Example trajectories of the state when out-group hate β is less than in-group love α. The trajectories over time (left column) and in the state space (middle column) show the evolution of $\theta(t) = [\theta^{\mathcal{B}}(t), \theta^{\mathcal{R}}(t)]$. The blue and red regions in middle column indicate regions where $\theta^{\mathcal{B}}(t), \theta^{\mathcal{R}}(t)$ increase (i.e., areas where $p_\theta^{\mathcal{B}}(0 \to 1) = 1$ and $p_\theta^{\mathcal{R}}(0 \to 1) = 1$ according to Eq. 4). The black arrows in the middle column indicate the path of the differential equation Eq. 4. The yellow arrows correspond to the time domain trajectory (left column). The figure shows how larger in-group love is necessary but not sufficient for the emergence of consensus. In particular, when the disparity between the sizes of the two groups is not too large compared to the disparity between α and β, consensus emerges.

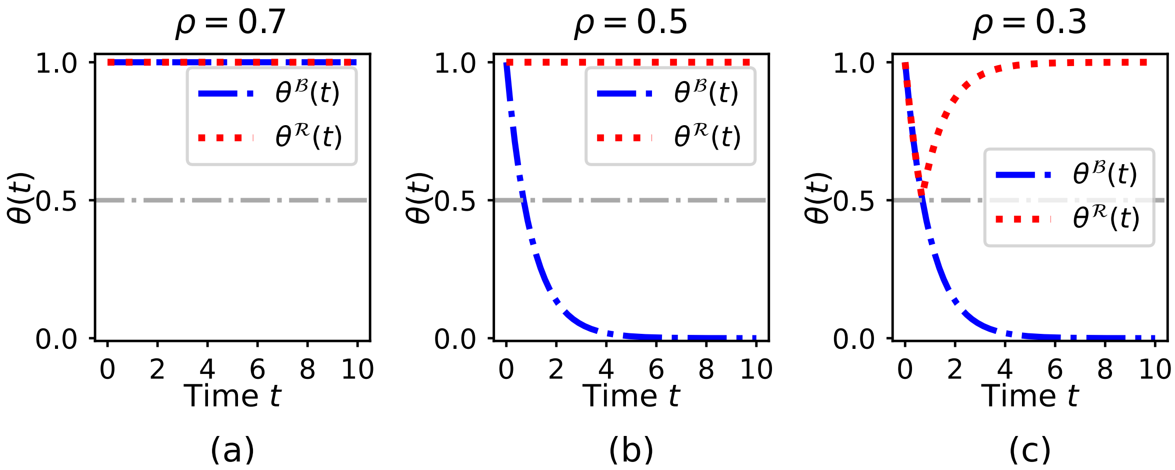

Figure S6: An illustration of how decreasing homophily can cause a party-line polarization from an initial state of global consensus. Figures correspond to $\alpha = 0.8, \beta = 0.7$ (larger in-group favoritism compared to out-group animosity) and $r = 0.65$ (a majority red group). Decreasing ρ from 0.7 (homophily) to 0.5 pushes the social network from Case-1 (consensus) to Case-3 (party-line polarization) in Fig. S1. Further decreasing ρ to 0.3 pushes the network to Case-4 which corresponds to an unstable state, where a small deviation leads to party-line polarization.

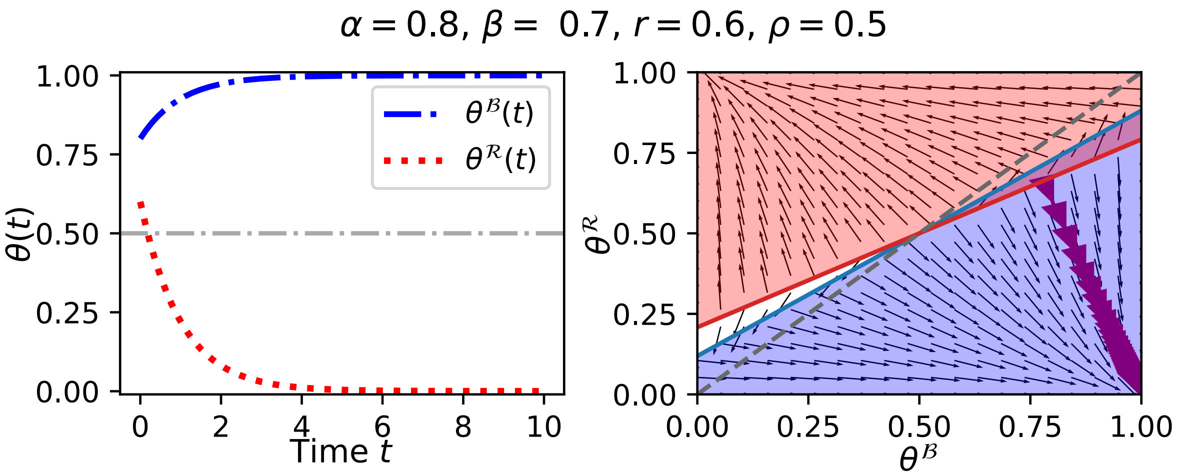

Figure S7: An illustration of a case where the two groups start with different popularity levels of the choices within them i.e., $\theta^{\mathcal{B}}(0) \neq \theta^{\mathcal{R}}(0)$, and the majority group adopts the choice that was initially less popular within it. The choice-1 is initially more popular within both groups with $\theta^{\mathcal{B}}(0) = 0.8, \theta^{\mathcal{R}}(0) = 0.6$. However, the majority red-group eventually adopts the choice that was initially less popular (i.e., choice-0 which had a 40% popularity) within it.

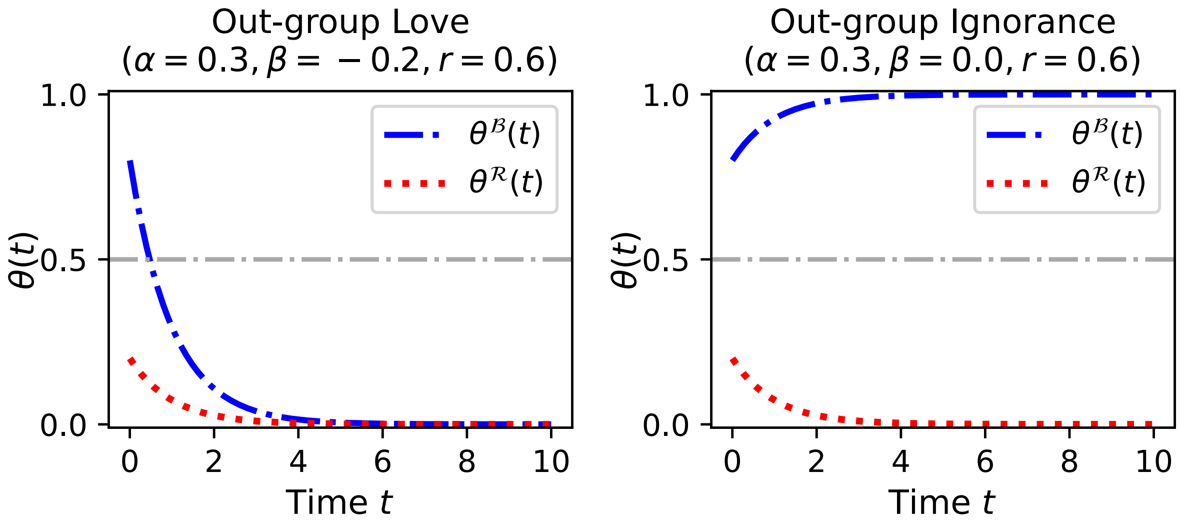

Figure S8: The figure shows an example of how out-group ignorance (right panel $\beta = 0$) can polarize a society compared to a case where there is out-group love (left panel $\beta < 0$). Thus, out-group ignorance has adverse implications compared to out-group love though it is better than out-group hate.

E Generalizations of the Model

E.1 Out-group Ignorance

The proposed model can be generalized to explore the implications of out-group ignorance where individuals neglect the out-group in their decisions (instead of actively trying to differ from it). As an example, Fig. S8 compares two cases related to out-group emotions (with all other parameters kept the same) using the model dynamics in Eq. 4: left panel shows a case where people look at the out-group positively and yet not as positively as their ingroup (i.e., $\alpha = 0.3, \beta = -0.2$) and the right panel shows a case where people completely ignore the out-group (i.e., $\alpha = 0.3, \beta = 0.0$). Starting from the same initial states, the out-group ignorance leads to party-line polarization and the out-group love (despite being smaller than in-group love) leads to consensus. Thus, the presence of even a slightly positive attitude towards the out-group (compared to the in-group) can facilitate consensus compared to out-group ignorance.

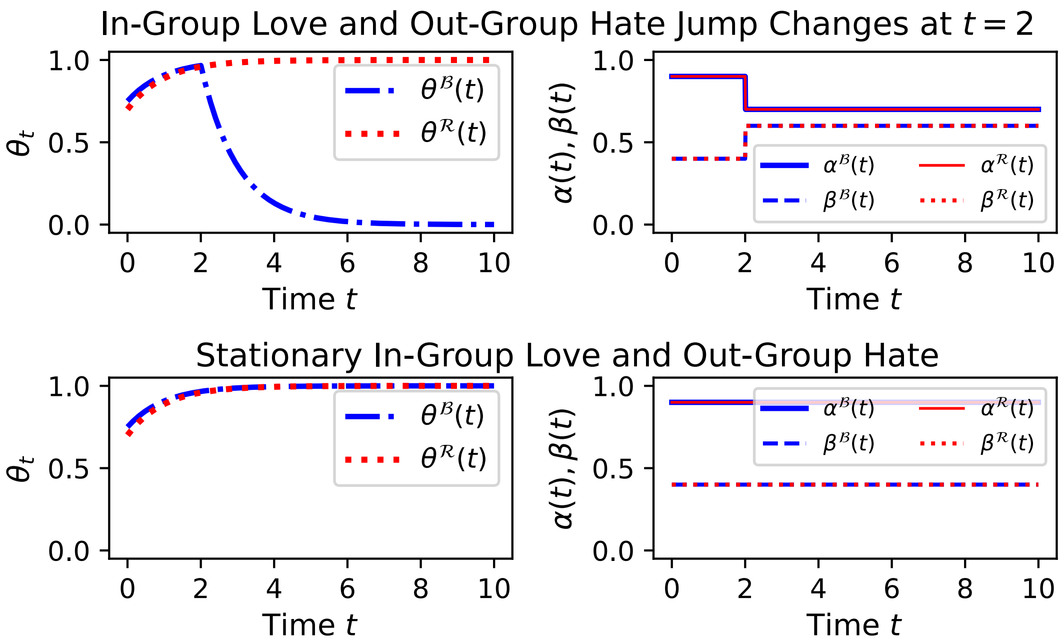

Figure S9: An example of how a sudden change in the in-group love and out-group hate (row 1) can lead to different dynamics compared to stationary parameters (row 2).

E.2 Group-dependent and Time-varying Parameters of Affective Polarization

It is possible to incorporate time-varying and/or state ($\theta(t)$)-dependent evolution of the in-group love and out-group hate. Below we show how they could be incorporated into the model with examples and further results.

To illustrate the effects of the dynamics of affective polarization parameters, we consider the more general case where in-group love and out-group hate depends on the party: $\alpha^{\mathcal{B}}(t), \beta^{\mathcal{B}}(t)$ (resp. $\alpha^{\mathcal{R}}(t), \beta^{\mathcal{R}}(t)$) denote the in-group love and out-group hate of the blue-group (resp. red-group) at time t.

- **Case 1: A sudden change in in-group love and out-group hate after a debate on a contentious issue or an event** (e.g., a supreme court ruling, an election, a change in social media platform policy, etc.).

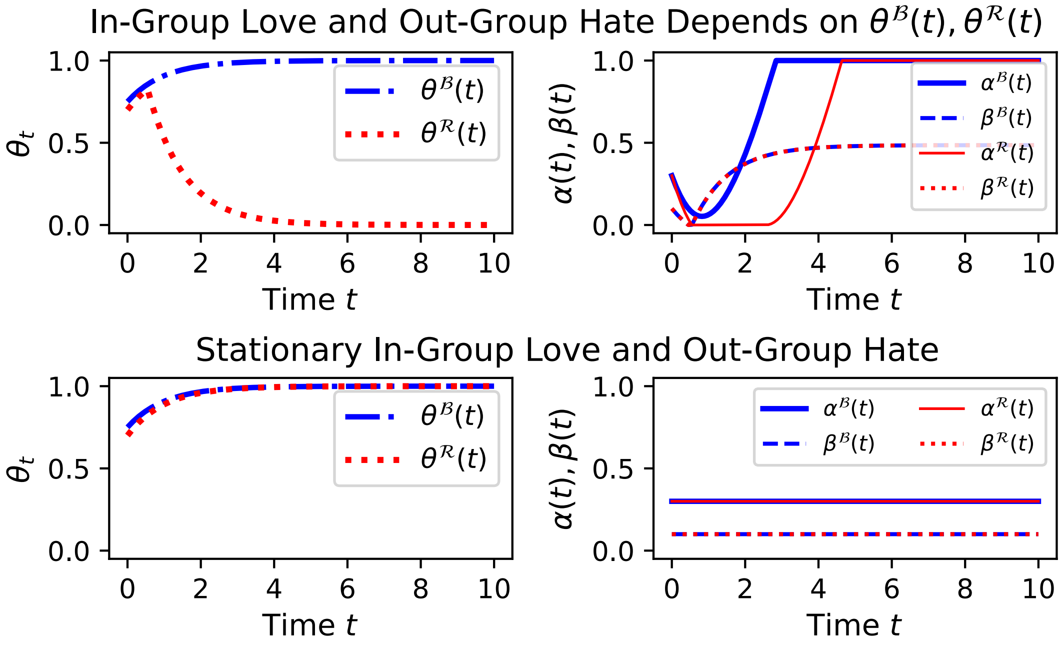

Figure S10: An example of how evolution of in-group love and out-group hate depending on the population state $\theta(t) = \left[\theta^{\mathcal{B}}(t), \theta^{\mathcal{R}}(t)\right]$ (row 1) can lead to different dynamics compared to stationary parameters (row 2).

This context can easily be incorporated into the model and explored when the timing and magnitude of the parameter change is known. This amounts to a jump change in the parameters of a dynamical system. Fig. S9 shows an example. In the first case (top row of Fig. S9), the in-group love decrease from 0.9 to 0.7 while out-group hate increases from 0.4 to 0.6. In the second case, (second row of Fig. S9), the in-group love and out-group hate remain fixed at 0.9 and 0.4, respectively. The system was initially heading towards consensus and would have achieved it (second row of Fig. S9) had there not been a sudden change of parameters, which led the population to a state of party-line polarization.

As the above example illustrates, such sudden changes in parameters and their implications can be explored via the proposed model.

- **Case 2: Evolution of the in-group love and out-group hate depending on the state**

$\theta(t) = \left[\theta^{\mathcal{B}}(t), \theta^{\mathcal{R}}(t)\right]$.

Different from the previous case, there can be more general scenarios where the evolution of in-group love and out-group hate depends on the population state $\theta(t)$. They can also be incorporated into the dynamics of the model by coupling the evolution of the party-dependent in-group love and out-group hate to the state $\theta(t) = \left[\theta^{\mathcal{B}}(t), \theta^{\mathcal{R}}(t)\right]$. To illustrate, we consider the following example context:

- *Evolution of In-group Love $\alpha^{\mathcal{B}}(t), \alpha^{\mathcal{R}}(t)$:* The rate of change of in-group love of a party depends on how cohesive the party is. For example, if most members of the blue-group have adopted a particular choice (e.g., masking), then there is high in-group cohesion in the blue group and the in-group love of the blue group $\alpha^{\mathcal{B}}(t)$ increases rapidly (till it reaches the maximum value of 1). On the other hand, if the blue group is largely split between the two choices (i.e., $\theta^{\mathcal{B}}(t)$ is closer to 0.5), then there is less in-group cohesion in-the blue group and the in-group love of the blue group $\alpha^{\mathcal{B}}(t)$ decreases rapidly (till it reaches the minimum value of 0). To formalize this, we use entropy of a Bernoulli random variable with parameter $\theta^{\mathcal{B}}(t)$. In particular, the discrete time stochastic dynamics corresponding to $\alpha^{\mathcal{B}}(t)$ can be written as,

$$\alpha^{\mathcal{B}}_{k+1} = \alpha^{\mathcal{B}}_{k} + \frac{1}{N}(1 - 2\text{Entropy}(\theta^{\mathcal{B}}_{k})).$$

  As shown in Fig. S11, $(1 - 2\text{Entropy}(\theta^{\mathcal{B}}_{k}))$ is negative when the blue group is split approximately equally between the two choices while it is positive when the blue group is cohesive. Therefore, this approach models the dynamics of $\alpha^{\mathcal{B}}(t)$. The dynamics of the out-group hate of the red-group can be modeled similarly.

- *Evolution of Out-group Hate $\beta^{\mathcal{B}}(t), \beta^{\mathcal{R}}(t)$:* The rate of change of out-group hate depends on the whether the two parties are heading towards the same or opposite di-

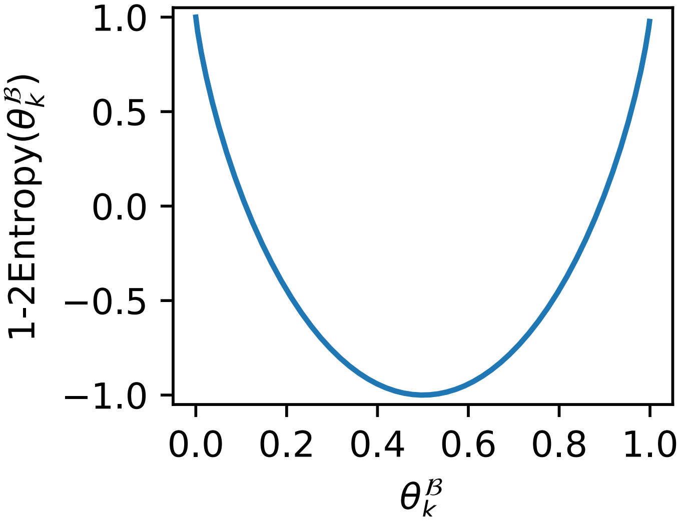

Figure S11: The variation of $1 - 2\text{Entropy}(\theta_k^{\mathcal{B}}))$ as a function of $\theta_k^{\mathcal{B}}$. This function is used to model the dynamics of the in-group love in the example setting we consider.

rection in terms of adopting a choice. For example, the out-group hate will increase if one group is rapidly adopting masking while the other is rapidly giving up masking. On the other hand, if both groups are adopting masking, then the out-group hate will decrease due to the similarity of the trend. This can be incorporated into the model for the blue group as

$$\beta_{k+1}^{\mathcal{B}} = \beta_k^{\mathcal{B}} + \frac{|\dot{\theta}^{\mathcal{B}}| + |\dot{\theta}^{\mathcal{R}}|}{2N} \left(\mathbb{1}\left\{ \text{sign}\left(\dot{\theta}^{\mathcal{B}}\right) \neq \text{sign}\left(\dot{\theta}^{\mathcal{R}}\right) \right\} - \mathbb{1}\left\{ \text{sign}\left(\dot{\theta}^{\mathcal{B}}\right) = \text{sign}\left(\dot{\theta}^{\mathcal{R}}\right) \right\} \right).$$

In particular the magnitude of the rate of change of the out-group hate is the average of the absolute values of the rates of the the states of the blue and red groups. The direction of the rate of change of the out-group hate is determined by whether the two groups are moving in the same or opposite directions. For the red-group, a similar argument holds.

Fig. S10 shows an example of these dynamics. Comparing row 1 (population state dependent parameter evolution) with the baseline in row 2 (time independent parameters)

illustrates how the dynamics of parameters can alter the trajectory of the population state.

- **Case 3: Evolution on α, β on a slower time scale (compared to $\theta(t)$).** When the parameters α, β evolve on a slower time scale compared to the population state $\theta(t)$, we can treat α, β as fixed parameters for the purpose of analyzing the dynamics of $\theta(t)$. The main results of our paper where we have treated α, β as fixed parameters apply in this setting for smaller time intervals.

As outlined above, the model and its analysis can be generalized to include the dynamics of the parameters of affective polarization in several ways.
